# Supplementary material for: SpRY greatly expands the genome editing scope in rice with highly flexible PAM recognition
Source: Genome Biol. 2021 Jan 4;22:6. doi: 10.1186/s13059-020-02231-9 (PMC7780387; doi:10.1186/s13059-020-02231-9)
Supplement: Supplementary file 1 — Additional file 1. Supplemental figures 1-18 and Supplementary Tables 1-3. [file 13059_2020_2231_MOESM1_ESM.pdf]

a

***OsPAL5* sgRNA1 PAM: NGG / SpG**

```
        5′-GGAGGAAUUCAGGAUCCUGA-3′
    5′-AAGAAAGGGAGGAATTCAGGATCCTGAAGGCTTCT-3′   WT
#2  5′-AAGAAAGGGAGGAATTCAGGAT---GAAGGCTTCT-3′   -3
    5′-AAGAAAGGGAGGAATTCAGGAT-CTGAAGGCTTCT-3′   -1
#11 5′-AAGAAAGGGAGGAA--------------GGCTTCT-3′   -14
    5′-AAGAAAGGGAGGAATTCAGGA------AGGCTTCT-3′   -6
#21 5′-AAGAAAGGGAGGAATTCAGGATCCTGAAGGCTTCT-3′   WT
    5′-AAGAAAGGGA----------------AAGGCTTCT-3′   -16
```

b

***OsGSK4* sgRNA1 PAM: NGG / SpG**

```
       5′-GUUGUUGGAACUGGAUCCUU-3′
    5′-GAACGAGTTGTTGGAACTGGATCCTTTGGTGTTGT-3′   WT
#1  5′-GAACGAGTTGTTGGAACTGGATCCTTTGGTGTTGT-3′   WT
    5′-GAACGAGTTGTTGGAACTGG---------TGTTGT-3′   -9
#10 5′-GAACGAGTTGTTGGAACTGGATCCTTTGGTGTTGT-3′   WT
    5′-GAACGAGTTGTTGGAACTGGATC-TTTGGTGTTGT-3′   -1
#16 5′-GAACGAGTTGTTGGAACTGGATCCTTTGGTGTTGT-3′   WT
    5′-GAACGAGTTGTTGGAACTGGATCtTTTGGTGTTGT-3′   -1/+1
```

c

***OsCERK1* sgRNA1 PAM: NGA / SpG**

```
        3′-GGCCUAGGGUUCCUUCCGGG-5′
    5′-CAACTCGCCGGATCCCAAGGAAGGCCTTAGGACG-3′    WT
#20 5′-CAACTCGCCGGATCCCAAGGAAGGCCTTAGGACG-3′    WT
    5′-CAACTCGCCG--TCCCAAGGAAGGCCTTAGGACG-3′    -2
#21 5′-CAACTCGCCGGATCCCAAGGAAGGCCTTAGGACG-3′    WT
    5′-CAACTCGCCccGGATCCCAAGGAAGGCCTTAGGACG-3′  +2
#24 5′-CAACTCGCCG-ATCCCAAGGAAGGCCTTAGGACG-3′    -1
    5′-CAACTCGCCGcGATCCCAAGGAAGGCCTTAGGACG-3′   +1
```

d

***OsGSK4* sgRNA2 PAM: NGT / SpG**

```
           3′-CCUAGGAAACCACAACAUAG-5′
    5′-GTTGGAACTGGATCCTTTGGTGTTGTATTCCAGGT-3′    WT
#3  5′-GTTGGAACTGGA-CCTTTGGTGTTGTATTCCAGGT-3′    -1
    5′-GTTGGAACTGGAtTCCTTTGGTGTTGTATTCCAGGT-3′   +1
#21 5′-GTTGGAACTGGATCCTTTGGTGTTGTATTCCAGGT-3′    WT
    5′-GTTGGAACTGGAaTCCTTTGGTGTTGTATTCCAGGT-3′   +1
#25 5′-GTTGGAACTGGAT---TTGGTGTTGTATTCCAGGT-3′    -3
    5′-GTTGGAACTGGAT-----GGTGTTGTATTCCAGGT-3′    -5
```

e

***OsETR2* sgRNA1 PAM: NGT / SpG**

```
        5′-GUCCCAUCUCUGUGGAUCCG-3′
    5′-GCCACCGATCCCATCTCTGTGGATCCGCGTCTTCT-3′   WT
#5  5′-GCCACCGATCCCATCTCTGTGGATCCGCGTCTTCT-3′   WT
    5′-GCCACCGATCCCATCTC--------CGCGTCTTCT-3′   -8
```

f

***OsCERK1* sgRNA2 PAM: NGC / SpG**

```
           3′-CCUAGGGUUCCUUCCGGAAG-5′
    5′-CAACTCGCCGGATCCCAAGGAAGGCCTTAGGACGT-3′    WT
#1  5′-CAACTCGCCGGATCCCAAGGAAGGCCTTAGGACGT-3′    WT
    5′-CAACTCGCC---TCCCAAGGAAGGCCTTAGGACGT-3′    -3
#4  5′-CAACTCGCCGGATCCCAAGGAAGGCCTTAGGACGT-3′    WT
    5′-CAACTCGCCGGATCCCAAGGAAgGGCCTTAGGACGT-3′   +1
#13 5′-CAACTCGCCGGAaTCCCAAGGAAGGCCTTAGGACGT-3′   +1
    5′-CAACTCGCCGGAaTCCCAAGGAAGGCCTTAGGACGT-3′   +1
```

g

***OsRLCK185* sgRNA1 PAM: NGC / SpG**

```
          3′-CACGUGUUUAACUCCGUGCG-5′
    5′-GCTTGGCAGTGCACAAATTGAGGCACGATGGCACA-3′    WT
#23 5′-GCTTGGCAGTGCACAAATTGAGGCACGATGGCACA-3′    WT
    5′-GCTTGGCAGTGaCACAAATTGAGGCACGATGGCACA-3′   +1
```

Figure S1. Analysis of SpG nuclease activity on different NGN PAMs in transgenic rice. **a-g** Representative mutant alleles of *OsPAL5* (**a**) and *OsGSK4* (**b**) with NGG PAMs, *OsCERK1* with an NGA PAM (**c**), *OsGSK4* (**d**) and *OsETR2* (**e**) with NGT PAMs, *OsCERK1* (**f**) and *OsRLCK185* (**g**) with an NGC PAM edited by SpG nuclease in T0 transgenic rice callus lines. WT, wild type; The PAM sequences and target sequences are highlighted in green and bold, respectively; nucleotide deletions and insertions are indicated by dashes and lowercase letters, respectively.

a

***OsPAL5* sgRNA1 PAM: NGG / SpCas9-NG**

```
            5′-GGAGGAAUUCAGGAUCCUGA-3′
      5′-AAGAAAGGGAGGAATTCAGGATCCTGAAGGCTTCT-3′    WT
#4  { 5′-AAGAAAGGGAGGAATTCAGG----TGAAGGCTTCT-3′    -4
    { 5′-AAGAAAGGGAGGAATTCAGGATC-TGAAGGCTTCT-3′    -1
#9  { 5′-AAGAAAGGGAGGAATTCAGG----TGAAGGCTTCT-3′    -4
    { 5′-AAGAAAGGGAGGAATTCAG--aatcGAAGGCTTCT-3′    -6/+4
#38 { 5′-AAGAAAGGGAGGAATTCAGGATCCTGAAGGCTTCT-3′    WT
    { 5′-AAGAAAGGGAGGAATTCAGGATC-TGAAGGCTTCT-3′    -1
```

b

***OsGSK4* sgRNA1 PAM: NGG / SpCas9-NG**

```
          5′-GUUGUUGGAACUGGAUCCUU-3′
      5′-GAACGAGTTGTTGGAACTGGATCCTTTGGTGTTGT-3′    WT
#4  { 5′-GAACGAGTTGTTGGAACTGGATCCTTTGGTGTTGT-3′    WT
    { 5′-GAACGAGTTGTTGGAACTGGATC-TTTGGTGTTGT-3′    -1
#32 { 5′-GAACGAGTTGTTGGAACTGGATCCTTTGGTGTTGT-3′    WT
    { 5′-GAACGAGTTGTTGGAACTGGA-CCTTTGGTGTTGT-3′    -1
#35 { 5′-GAACGAGTTGTTGGAACTGGATCCTTTGGTGTTGT-3′    WT
    { 5′-GAACGAGTTGTTGGAACTGGAT----TGGTGTTGT-3′    -4
```

c

***OsCERK1* sgRNA1 PAM: NGA / SpCas9-NG**

```
            3′-GGCCUAGGGUUCCUUCCGGG-5′
      5′-CAACTCGCCGGATCCCAAGGAAGGCCTTAGGAC-3′      WT
#2  { 5′-CAACTCGCCG-ATCCCAAGGAAGGCCTTAGGAC-3′      -1
    { 5′-CAACTCGCCGtGATCCCAAGGAAGGCCTTAGGAC-3′     +1
#4  { 5′-CAACTCGCCG-ATCCCAAGGAAGGCCTTAGGAC-3′      -1
    { 5′-CAACTCGCCGcGATCCCAAGGAAGGCCTTAGGAC-3′     +1
#13 { 5′-CAACTCGCCGGATCCCAAGGAAGGCCTTAGGAC-3′      WT
    { 5′-CAACTCGCCG-ATCCCAAGGAAGGCCTTAGGAC-3′      -1
```

d

***OsGSK4* sgRNA2 PAM: NGT / SpCas9-NG**

```
                3′-CCUAGGAAACCACAACAUAG-5′
      5′-GTTGGAACTGGATCCTTTGGTGTTGTATTCCAGGT-3′    WT
#1  { 5′-GTTGGAACTGGAaTCCTTTGGTGTTGTATTCCAGGT-3′   +1
    { 5′-GTTGGAACTGGAgTCCTTTGGTGTTGTATTCCAGGT-3′   +1
#4  { 5′-GTTGGAACTGGATCCTTTGGTGTTGTATTCCAGGT-3′    WT
    { 5′-GTTG---------------------TATTCCAGGT-3′    -21
#13 { 5′-GTTGGAACTGGAT---TTGGTGTTGTATTCCAGGT-3′    -3
    { 5′-GTTGGAACTGG-----TTGGTGTTGTATTCCAGGT-3′    -5
```

e

***OsETR2* sgRNA1 PAM: NGT / SpCas9-NG**

```
              5′-GUCCCAUCUCUGUGGAUCCG-3′
      5′-GCCACCGATCCCATCTCTGTGGATCCGCGTCTTCT-3′    WT
#9  { 5′-GCCACCGATCCCATCTCTGTGGATCCGCGTCTTCT-3′    WT
    { 5′-GCCACCGATCCCATCTCTGTG---CCGCGTCTTCT-3′    -3
#14 { 5′-GCCACCGATCCCATCTCTGTGGATCCGCGTCTTCT-3′    WT
    { 5′-GCCACCGATCCCATCTCTGTGGATtCCGCGTCTTCT-3′   +1
```

f

***OsCERK1* sgRNA2 PAM: NGC / SpCas9-NG**

```
                3′-CCUAGGGUUCCUUCCGGAAG-5′
      5′-CAACTCGCCGGATCCCAAGGAAGGCCTTAGGACGT-3′    WT
#3  { 5′-CAACTCGCCGGATCCCAAGGAAGGCCTTAGGACGT-3′    WT
    { 5′-CAACTCGCCGGA-CCCAAGGAAGGCCTTAGGACGT-3′    -1
#4  { 5′-CAACTCGCCGGATCCCAAGGAAGGCCTTAGGACGT-3′    WT
    { 5′-CAACTCGCCGGA------GGAAGGCCTTAGGACGT-3′    -6
#7  { 5′-CAACTCGCCGGATCCCAAGGAAGGCCTTAGGACGT-3′    WT
    { 5′-CAACTCGCCGGATtCCCAAGGAAGGCCTTAGGACGT-3′   +1
```

g

***OsRLCK185* sgRNA1 PAM: NGC / SpCas9-NG**

```
               3′-CACGUGUUUAACUCCGUGCG-5′
      5′-GCTTGGCAGTGCACAAATTGAGGCACGATGGCACA-3′    WT
#1  { 5′-GCTTGGCAGTGCACAAATTGAGGCACGATGGCACA-3′    WT
    { 5′-GCTTGGCAGTGtCACAAATTGAGGCACGATGGCACA-3′   +1
```

Figure S2. Analysis of SpCas9-NG nuclease activity on different NGN PAMs in transgenic rice. **a-g** Representative mutant alleles of *OsPAL5* (**a**) and *OsGSK4* (**b**) with NGG PAMs, *OsCERK1* with an NGA PAM (**c**), *OsGSK4* (**d**) and *OsETR2* (**e**) with NGT PAMs, *OsCERK1* (**f**) and *OsRLCK185* (**g**) with an NGC PAM edited by SpCas9-NG nuclease in T0 transgenic rice callus lines. WT, wild type; The PAM sequences and target sequences are highlighted in green and bold, respectively; nucleotide deletions and insertions are indicated by dashes and lowercase letters, respectively.

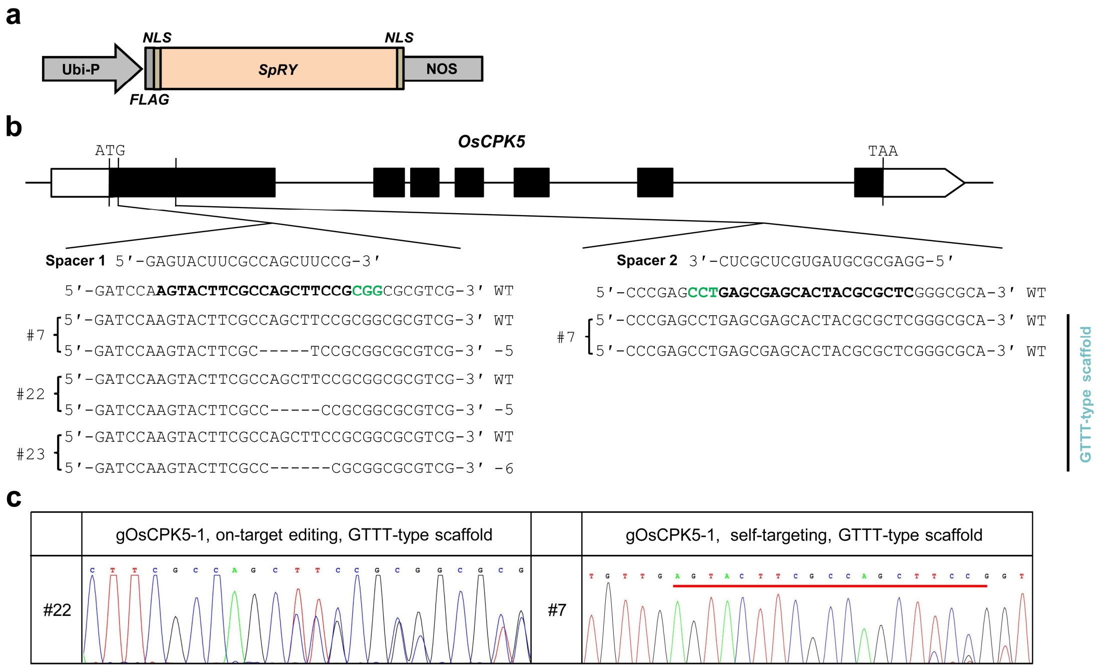

Figure S3. Targeted genome editing of *OsCPK5* by SpRY endonuclease in transgenic rice. **a** The gene construct of SpRY nuclease used for genome editing in transgenic rice. Ubi-P, maize ubiquitin 1 promoter; *NLS*, nuclear localization sequence. **b** Sequence results of the SpRY-edited *OsCPK5* at the NGG PAM sites using GTTT-type *sgRNA* scaffold in T0 transgenic rice callus lines. The exons are indicated by the black boxes; The PAM sequences and target sequences are highlighted in green and bold, respectively; Nucleotide deletions and insertions are indicated by dashes and lowercase letters, respectively. **c** Representative sanger sequencing chromatograms of the mutant *OsCPK5* allele and *OsCPK5-sgRNA* transgene in independent transgenic lines. The target region is underlined.

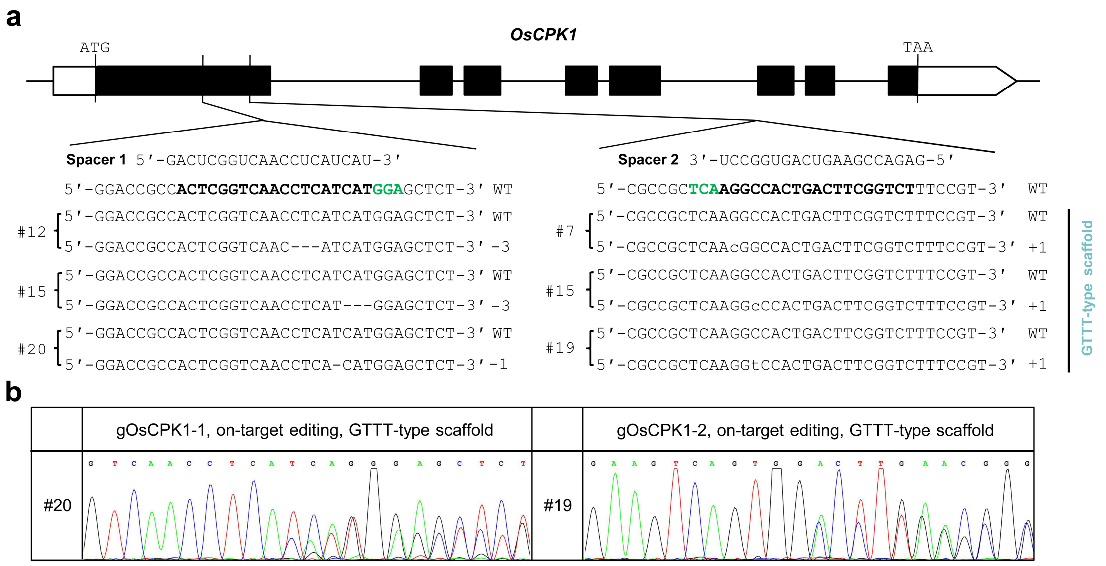

Figure S4. Targeted genome editing of *OsCPK1* by SpRY endonuclease in transgenic rice. **a** Sequence results of the SpRY-edited *OsCPK1* at the NGA PAM sites using GTTT-type *sgRNA* scaffold in T0 transgenic rice callus lines. The exons are indicated by the black boxes; The PAM sequences and target sequences are highlighted in green and bold, respectively; Nucleotide deletions and insertions are indicated by dashes and lowercase letters, respectively. **b** Representative sanger sequencing chromatograms of the mutant *OsCPK1* alleles in independent transgenic lines.

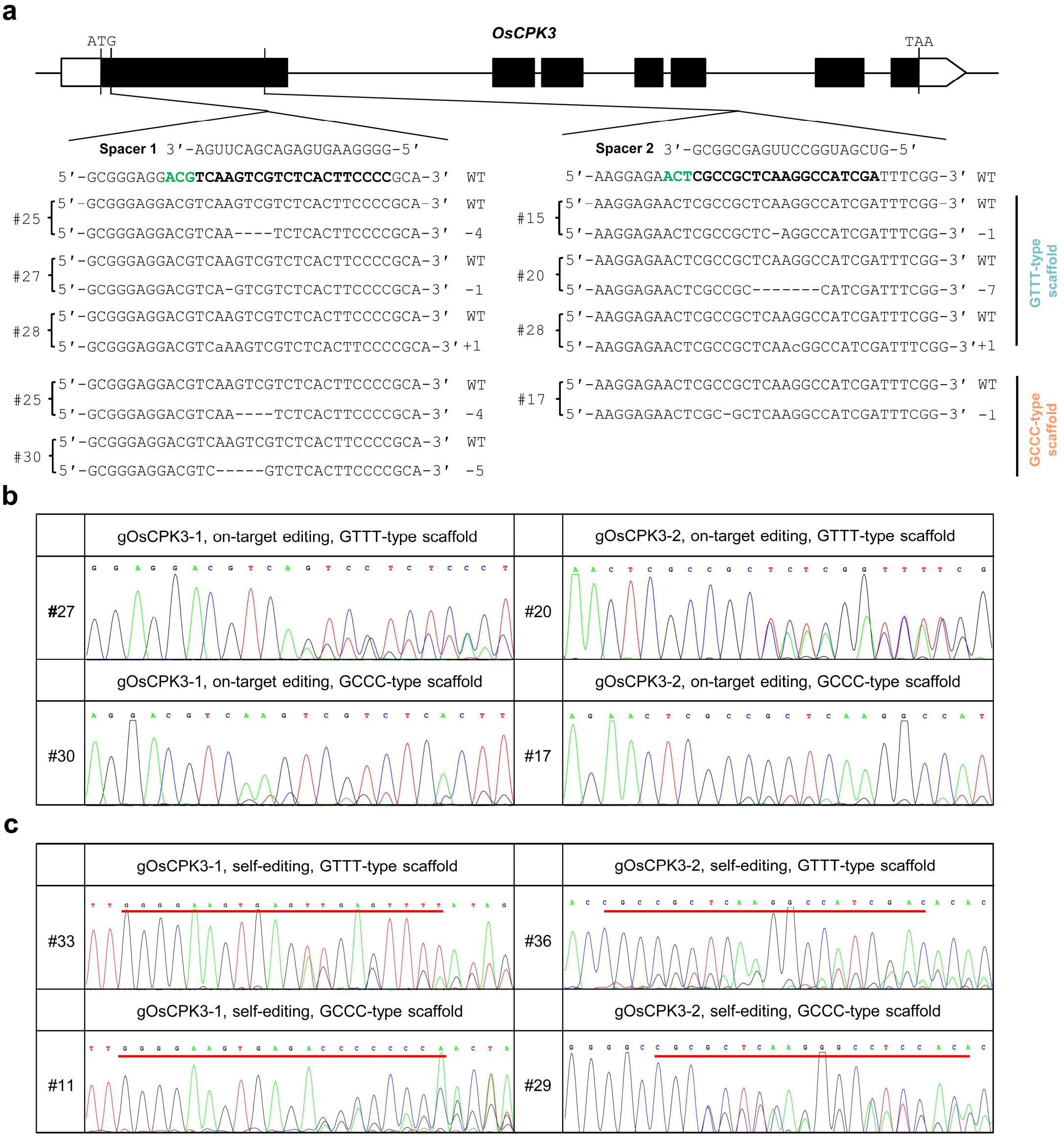

Figure S5. Targeted genome editing of *OsCPK3* by SpRY endonuclease in transgenic rice. **a** Sequence results of the SpRY-edited *OsCPK3* at the NGT PAM sites using GTTT-type and GCCC-type *sgRNA* scaffolds in T0 transgenic rice callus lines. The exons are indicated by the black boxes; The PAM sequences and target sequences are highlighted in green and bold, respectively; Nucleotide deletions and insertions are indicated by dashes and lowercase letters, respectively. **b** Representative sanger sequencing chromatograms of the mutant *OsCPK3* alleles in independent transgenic lines. **c** Representative sanger sequencing chromatograms of the mutant *OsCPK3-sgRNA* transgenes in independent transgenic lines. The target regions are underlined.

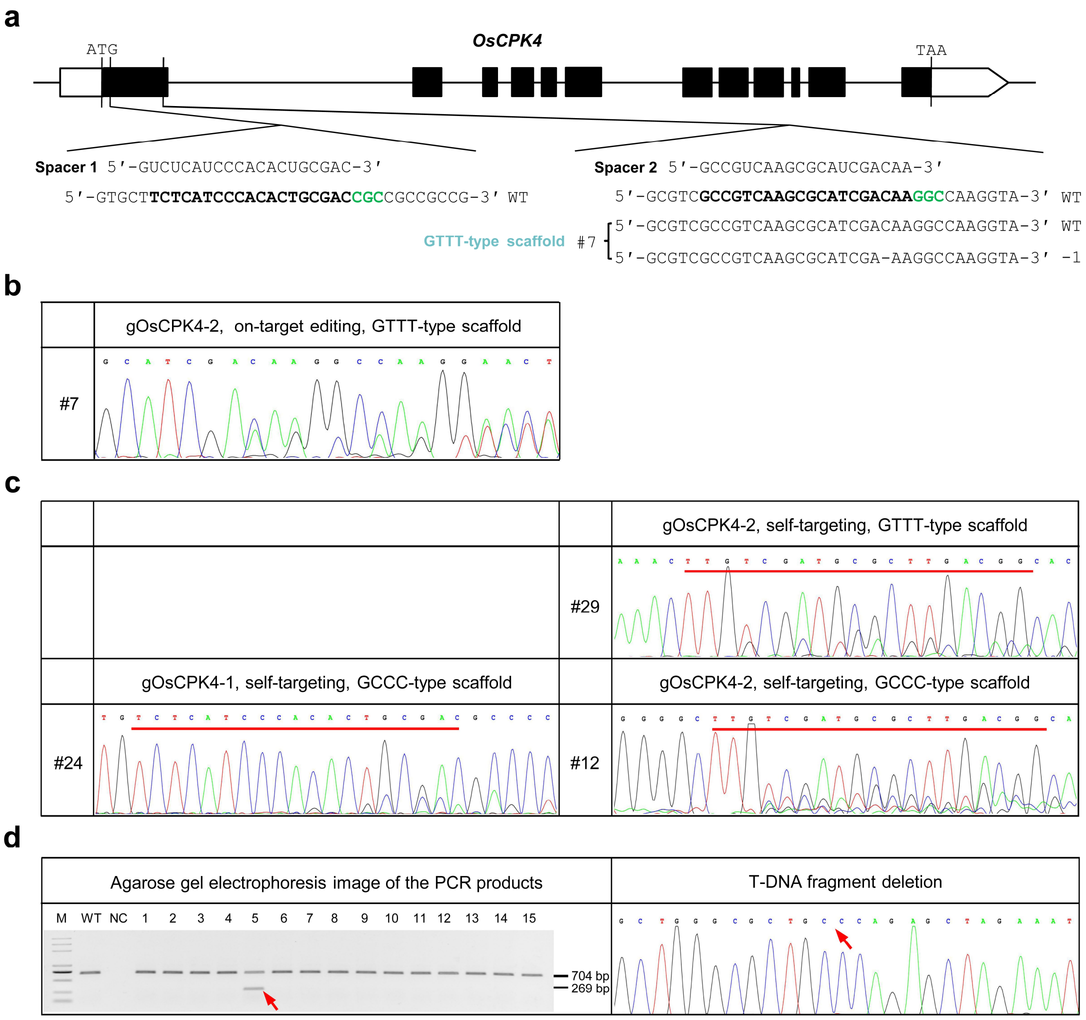

Figure S6. Targeted genome editing of *OsCPK4* by SpRY endonuclease in transgenic rice. **a** Sequence results of the SpRY-edited *OsCPK4* at the NGC PAM sites using GTTT-type *sgRNA* scaffold in T0 transgenic rice callus lines. The exons are indicated by the black boxes; The PAM sequences and target sequences are highlighted in green and bold, respectively; Nucleotide deletions and insertions are indicated by dashes and lowercase letters, respectively. **b** Representative sanger sequencing chromatogram of the mutant *OsCPK4* allele in independent transgenic line. **c** Representative sanger sequencing chromatograms of the mutant *OsCPK4-sgRNA* transgenes in independent transgenic callus lines. The target regions are underlined. **d** DNA fragment deletion between two *OsCPK4-sgRNA* transgenes induced by SpRY in transgenic lines. WT, wild type; NC, negative control. The deletion-junction site is marked by a red arrow.

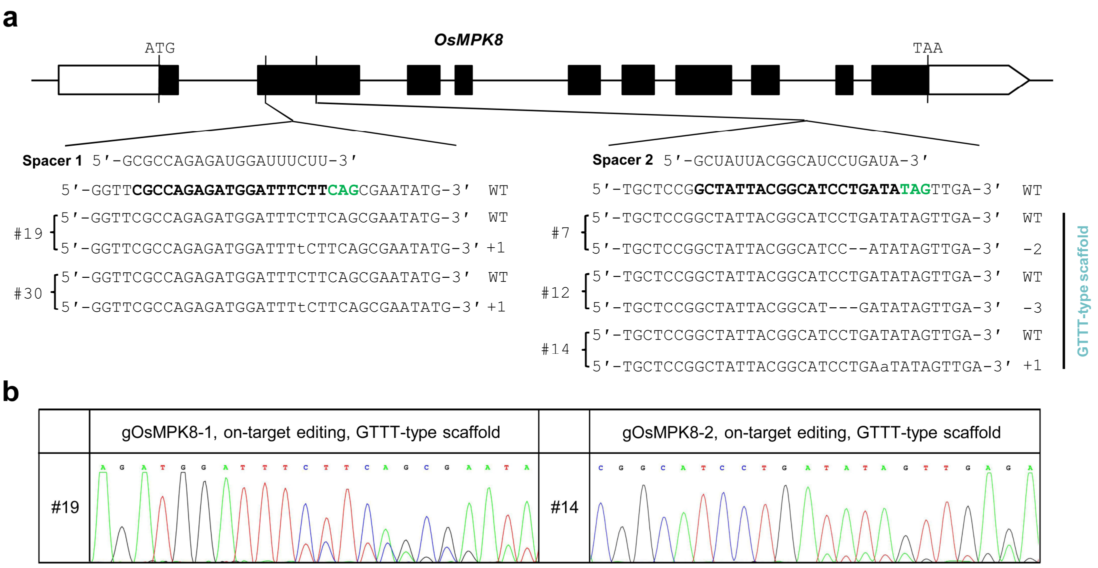

Figure S7. Targeted genome editing of *OsMPK8* by SpRY endonuclease in transgenic rice. **a** Sequence results of the SpRY-edited *OsMPK8* at the NAG PAM sites using GTTT-type *sgRNA* scaffold in T0 transgenic rice callus lines. The exons are indicated by the black boxes; The PAM sequences and target sequences are highlighted in green and bold, respectively; Nucleotide deletions and insertions are indicated by dashes and lowercase letters, respectively. **b** Representative sanger sequencing chromatograms of the mutant *OsMPK8* alleles in independent transgenic lines.

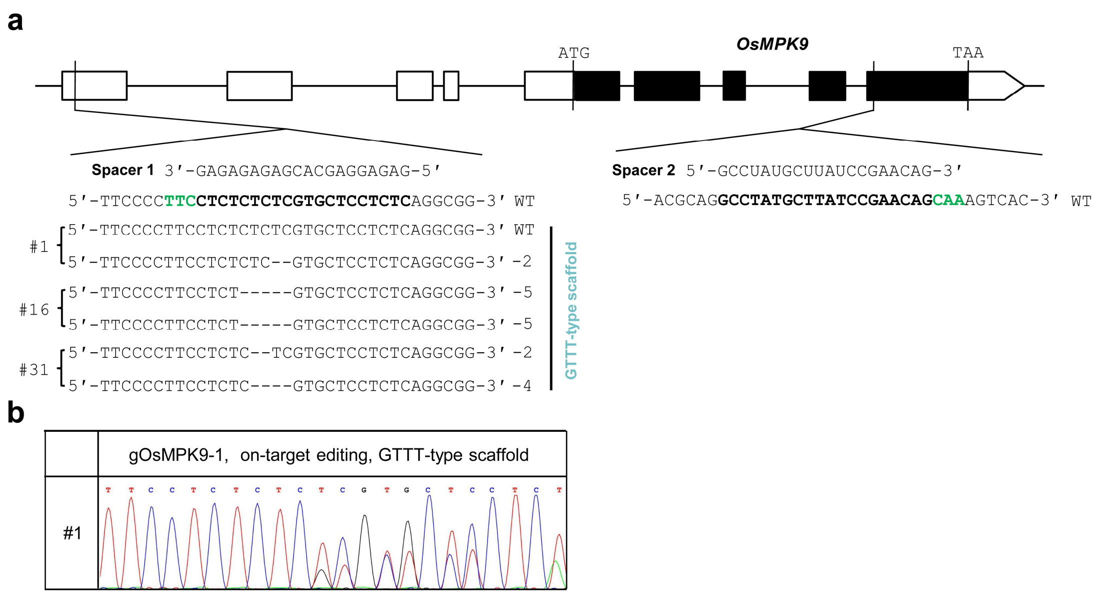

Figure S8. Targeted genome editing of *OsMPK9* by SpRY endonuclease in transgenic rice. **a** Sequence results of the SpRY-edited *OsMPK9* at the NAA PAM sites using GTTT-type *sgRNA* scaffold in T0 transgenic rice callus lines. The exons are indicated by the black boxes; The PAM sequences and target sequences are highlighted in green and bold, respectively; Nucleotide deletions and insertions are indicated by dashes and lowercase letters, respectively. **b** Representative sanger sequencing chromatogram of mutant *OsMPK9* allele in independent transgenic line.

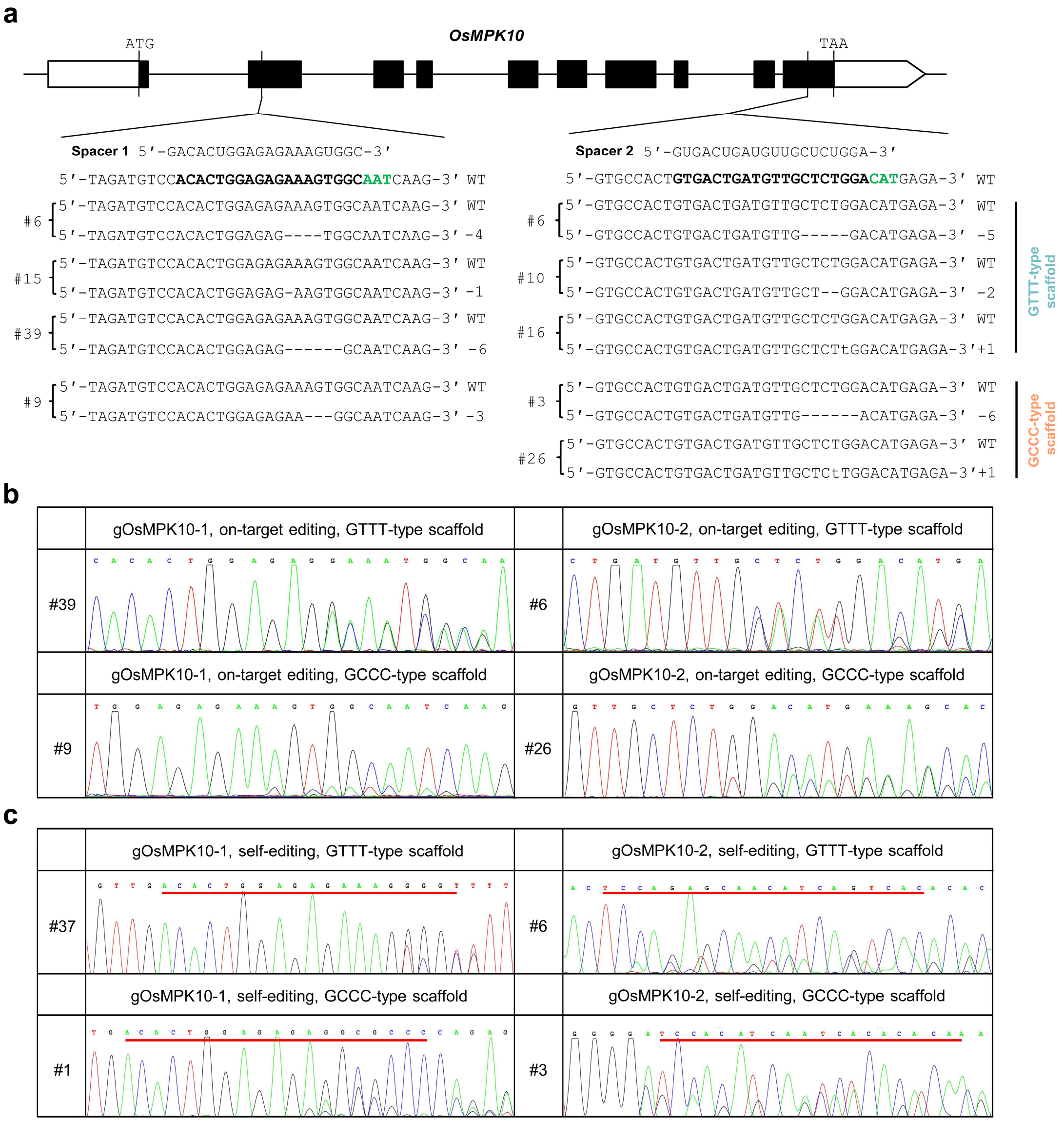

Figure S9. Targeted genome editing of *OsMPK10* by SpRY endonuclease in transgenic rice. **a** Sequence results of the SpRY-edited *OsMPK10* at the NAT PAM sites using GTTT-type and GCCC-type sgRNA scaffolds in T0 transgenic rice callus lines. The exons are indicated by the black boxes; The PAM sequences and target sequences are highlighted in green and bold, respectively; Nucleotide deletions and insertions are indicated by dashes and lowercase letters, respectively. **b** Representative sanger sequencing chromatograms of the mutant *OsMPK10* alleles in independent transgenic lines. **c** Representative sanger sequencing chromatograms of the mutant *OsMPK10-sgRNA* transgenes in independent transgenic lines. The target regions are underlined.

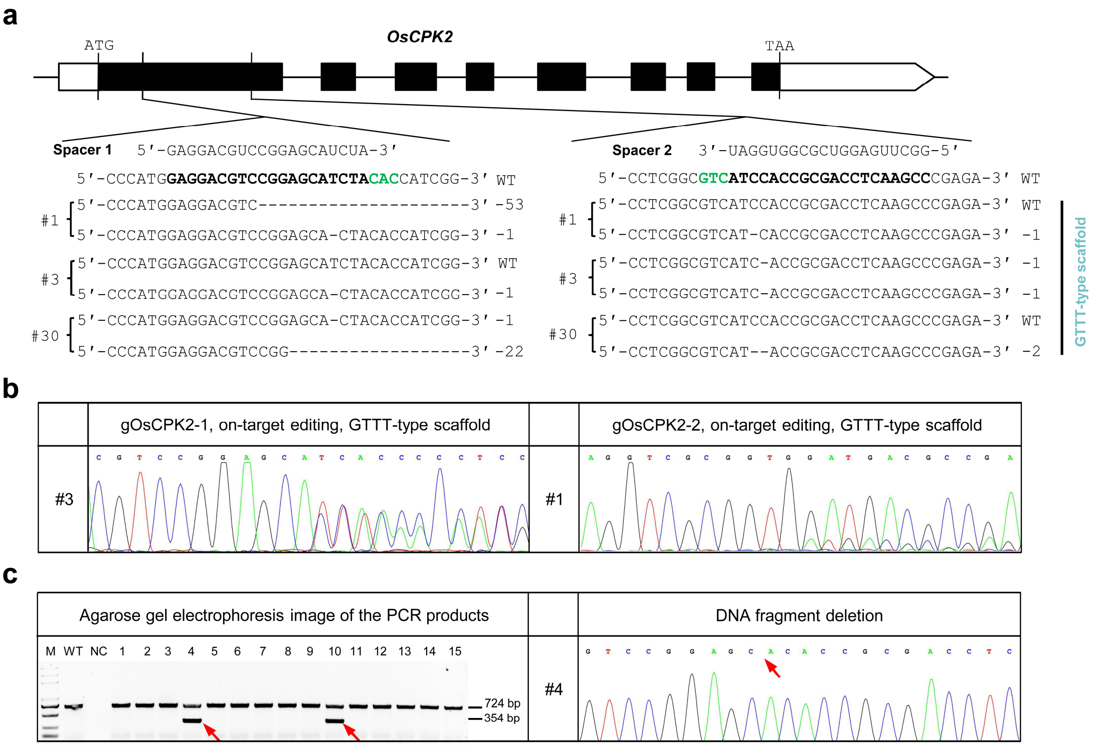

Figure S10. Targeted genome editing of *OsCPK2* by SpRY endonuclease in transgenic rice. **a** Sequence results of the SpRY-edited *OsCPK2* at the NAC PAM sites using GTTT-type sgRNA scaffold in T0 transgenic rice callus lines. The exons are indicated by the black boxes; The PAM sequences and target sequences are highlighted in green and bold, respectively; Nucleotide deletions and insertions are indicated by dashes and lowercase letters, respectively. **b** Representative sanger sequencing chromatograms of mutant *OsCPK2* alleles in independent transgenic lines. **c** DNA fragment deletion in *OsCPK2* induced by SpRY in transgenic line. WT, wild type; NC, negative control. The deletion-junction site is marked by a red arrow.

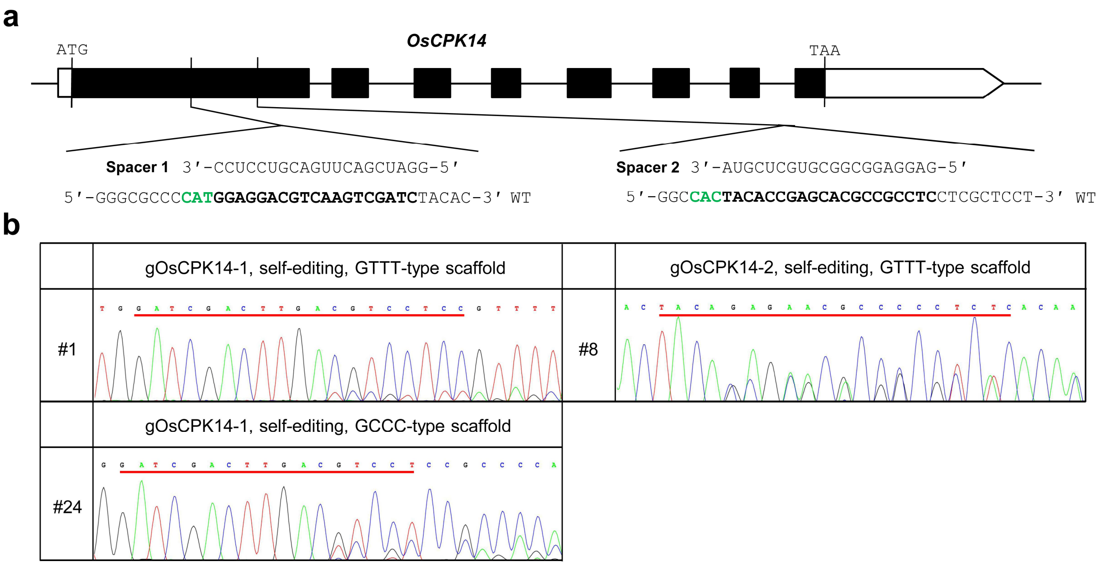

Figure S11. Targeted genome editing of *OsCPK14* by SpRY endonuclease in transgenic rice. **a** The target sites of *OsCPK14* in rice. The exons are indicated by the black boxes; The PAM sequences and target sequences are highlighted in green and bold, respectively. **b** Representative sanger sequencing chromatograms of the mutant *OsCPK14-sgRNA* transgenes in independent transgenic lines. The target regions are underlined.

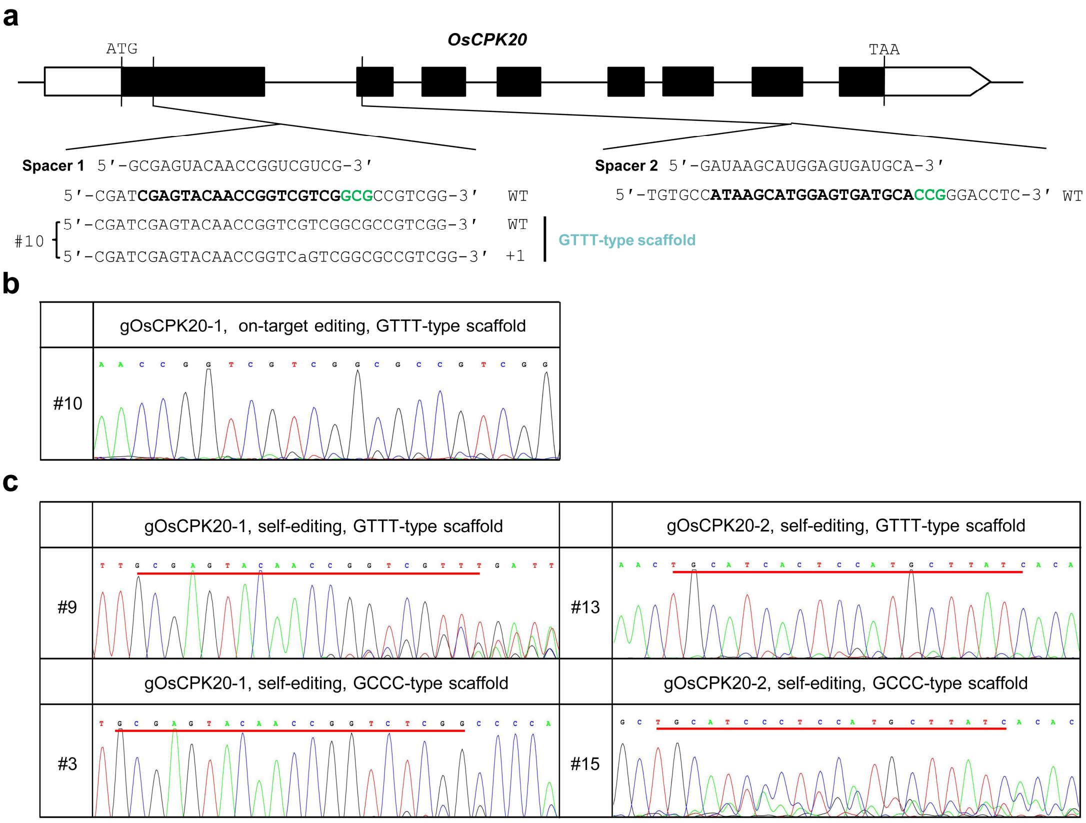

Figure S12. Targeted genome editing of *OsCPK20* by SpRY endonuclease in transgenic rice. **a** Sequence results of the SpRY-edited *OsCPK20* at the NCG PAM sites using GTTT-type *sgRNA* scaffold in T0 transgenic rice callus lines. The exons are indicated by the black boxes; The PAM sequences and target sequences are highlighted in green and bold, respectively; Nucleotide deletions and insertions are indicated by dashes and lowercase letters, respectively. **b** Representative sanger sequencing chromatogram of the mutant *OsCPK20* allele in independent transgenic line. **c** Representative sanger sequencing chromatograms of the mutant *OsCPK20-sgRNA* transgenes in independent transgenic lines. The target regions are underlined.

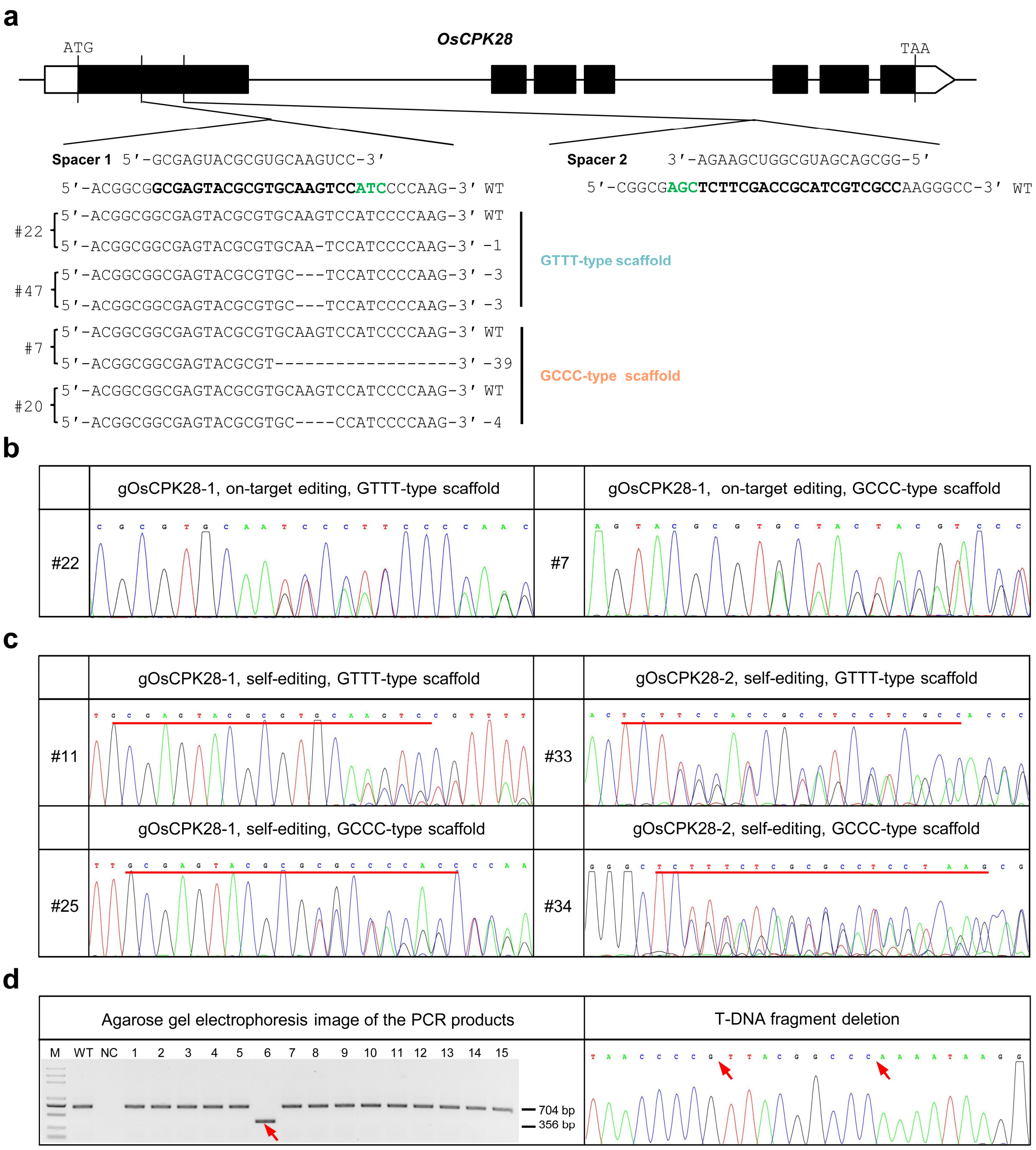

Figure S13. Targeted genome editing of *OsCPK28* by SpRY endonuclease in transgenic rice. **a** Sequence results of the SpRY-edited *OsCPK28* at the NTC and NCT PAM site using GTTT-type and GCCC-type sgRNA scaffolds in T0 transgenic rice callus lines. The exons are indicated by the black boxes; The PAM sequences and target sequences are highlighted in green and bold, respectively; Nucleotide deletions and insertions are indicated by dashes and lowercase letters, respectively. **b** Representative sanger sequencing chromatograms of the mutant *OsCPK28* alleles in independent transgenic lines. **c** Representative sanger sequencing chromatograms of the mutant *OsCPK28-sgRNA* transgenes in independent transgenic lines. The target regions are underlined. **d** DNA fragment deletion between two *OsCPK28-sgRNA* transgenes induced by SpRY in transgenic line. WT, wild type; NC, negative control. The deletion-junction site is marked by a red arrow.

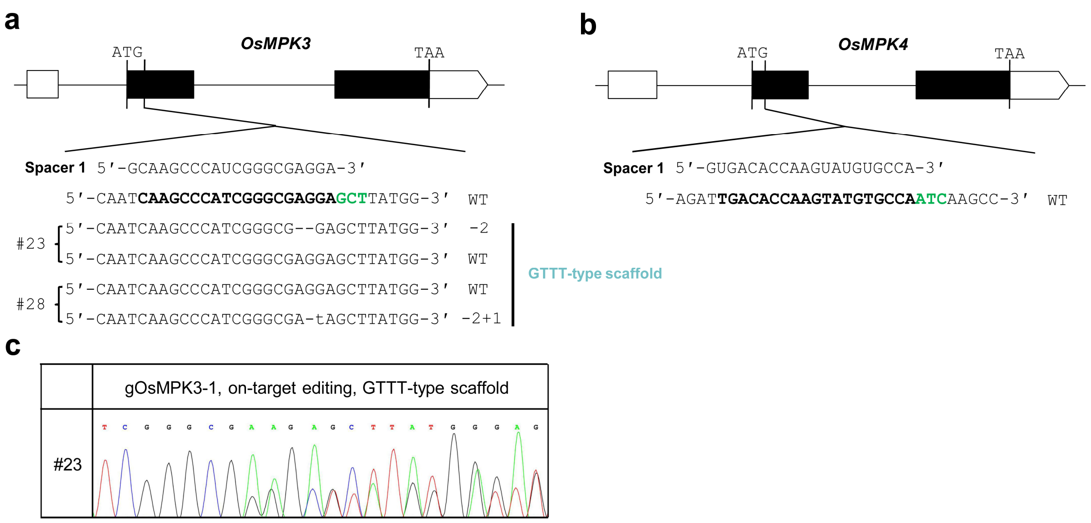

Figure S14. Targeted genome editing of *OsMPK3* and *OsMPK4* simultaneously by SpRY endonuclease in transgenic rice. **a** Sequence results of the SpRY-edited *OsMPK3* at the NCT PAM site using GTTT-type *sgRNA* scaffold in T0 transgenic rice callus lines. Nucleotide deletions and insertions are indicated by dashes and lowercase letters, respectively. **b** The target sites of *OsMPK4* in rice. In (**a** and **b**), the exons are indicated by the black boxes; The PAM sequences and target sequences are highlighted in green and bold, respectively; **c** Representative sanger sequencing chromatogram of mutant *OsMPK3* allele in transgenic line.

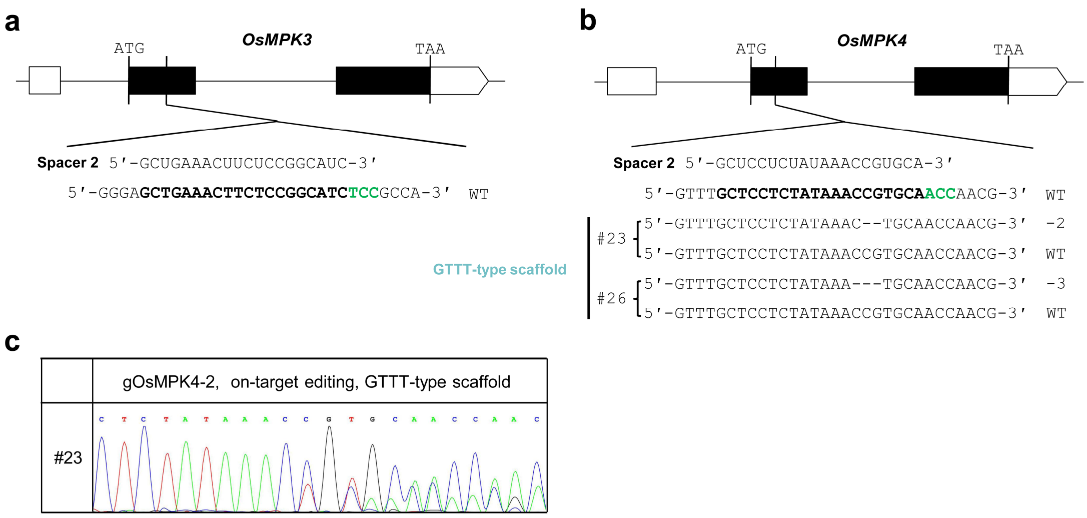

Figure S15. Targeted genome editing of *OsMPK3* and *OsMPK4* simultaneously by SpRY endonuclease in transgenic rice. **a** The target sites of *OsMPK3* in rice. **b** Sequence results of the SpRY-edited *OsMPK4* at the NCC PAM site using GTTT-type *sgRNA* scaffold in T0 transgenic rice callus lines. Nucleotide deletions and insertions are indicated by dashes and lowercase letters, respectively. In (**a** and **b**), the exons are indicated by the black boxes; The PAM sequences and target sequences are highlighted in green and bold, respectively; **c** Representative sanger sequencing chromatogram of mutant *OsMPK4* allele in transgenic line.

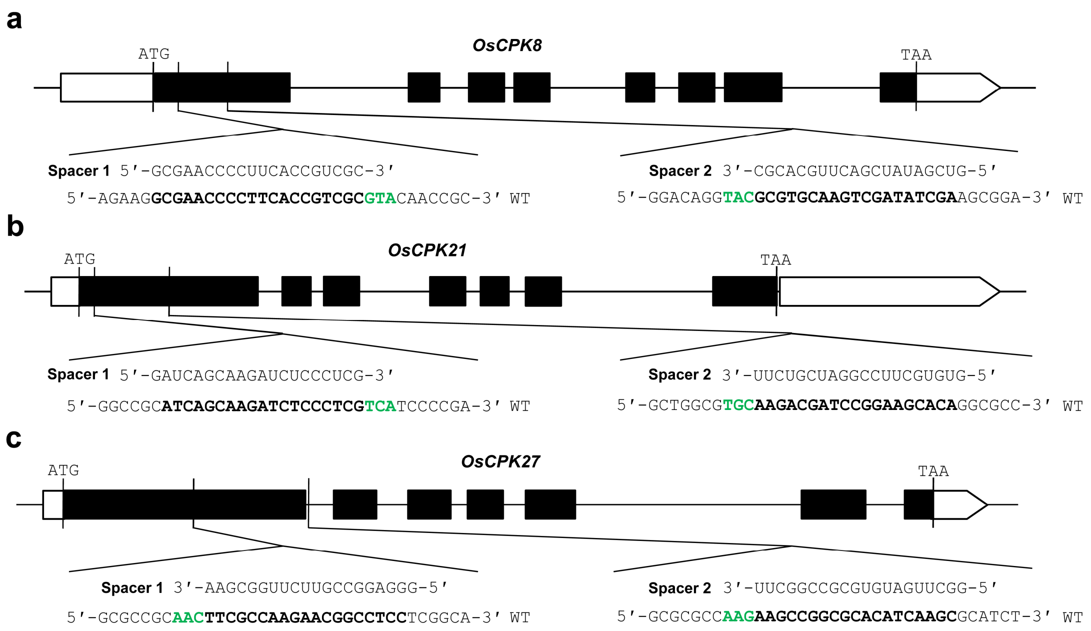

Figure S16. Targeted genome editing of *OsCPK8, OsCPK21,* and *OsCPK27* by SpRY endonuclease in transgenic rice. **a-c** The target sites of *OsCPK8* (**a**)*, OsCPK21* (**b**)*,* and *OsCPK27* (**c**) in rice. The exons are indicated by the black boxes; The PAM sequences and target sequences are highlighted in green and bold, respectively; Nucleotide deletions and insertions are indicated by dashes and lowercase letters, respectively.

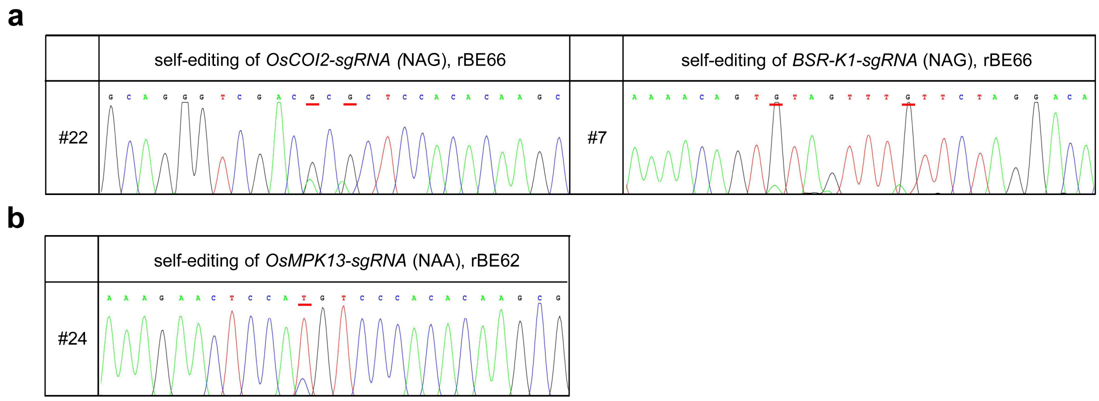

Figure S17. Self-editing activity of rBE66 and rBE62 in transgenic rice. **a** Representative sanger sequencing chromatograms of the *OsCOI2-sgRNA* and *BSR-K1-sgRNA* transgenes carrying nucleotide substitutions induced by rBE66 in independent transgenic callus lines. **b** Representative sanger sequencing chromatograms of the *OsMPK13-sgRNA* transgene carrying nucleotide substitution induced by rBE62 in independent transgenic callus line. In (**a** and **b**), the nucleotide substitutions are underlined.

a

***OsMPK8*-targeting sgRNA 1**

	Target region	Gene	Freq.
On-target	CGCCAGAGATGGATTTCTTCAG	*Os01g0665200/OsMPK8*	5.71%
Off-target 1	CAACAGAGATGGATTTCTTCAG	*Os05g0566450*	0.00%

***OsMPK10*-targeting sgRNA 2**

	Target region	Gene or Position	Freq.
On-target	GTGACTGATGTTGCTCTGGACAT	*Os01g0629900/OsMPK10*	43.58%
Off-target 1	ACGGCTGATGTTGCTCTGGACAT	*Os05g0582450*	0.00%
Off-target 2	CTGACTGATGTTGATCTGGTGGT	Chr12:10762404-2382	0.00%
Off-target 3	GGGACAGATGTTGCTCTGGAATT	Chr08:17339974-9952	0.00%

***OsCPK3*-targeting sgRNA 2**

	Target region	Gene	Freq.
On-target	TCGATGGCCTTGAGCGGCGAGT	*Os01g0832300/OsCPK3*	20.00%
Off-target 1	TCGATGGCCTTGAGCGGCGCGT	*Os04g0560600/OsCPK12*	0.00%
Off-target 2	TCGATGACCTTGAGCGGCGAGT	*Os08g0540400/OsCPK21*	15.00%
Off-target 3	TCAGTGGCCTTGAGCGGCGAGT	*Os01g0622600/OsCPK1*	0.00%
Off-target 4	TCGATGGCCTTGAGCTGCGCGT	*Os03g0688300/OsCPK9*	0.00%
Off-target 5	TCGATGGTCTTGAGCGGCGCGT	*Os12g0486600/OsCPK27*	0.00%
Off-target 6	TCGGTGGCCTTGAGCGGCGCGT	*Os11g0136600/OsCPK25*	2.50%

***OsCPK28*-targeting sgRNA 1**

	Target region	Gene	Freq.
On-target	GCGAGTACGCGTGCAAGTCCATC	*Os12g0169800/OsCPK28*	73.91%
Off-target 1	GCGAGTTCGCGTGCAAGTCCATC	*Os11g0171500/OsCPK24*	0.00%
Off-target 2	GCGAGTACGCGTGCAAGTCGATC	*Os12g0486600/OsCPK27*	0.00%

***OsCPK28*-targeting sgRNA 2**

	Target region	Gene	Freq.
On target1	AGCTCTTCGACCGCATCGTCGCC	*Os12g0169800/OsCPK28*	0.00%
Off-target 1	AGCTCTTCGACCGCATCGTCGCC	*Os11g0171500/OsCPK24*	0.00%
Off-target 2	AGCTCTTCGACCGCATCGTCGCC	*Os12g0230200/OsCPK29*	6.52%
Off-target 3	AGCTGTTCGACCGCATCGTCGCC	*Os05g0467150*	0.00%
Off-target 4	AGCTCTTCGACCGCATCATCGCC	*Os05g0585500/OsCPK15*	0.00%
Off-target 5	AGCTCTTCGACCGCATCTTCGCC	*Os09g0514200/OsCPK22*	0.00%
Off-target 6	AGCTCTTCGACCGGATCGTCGCC	*Os07g0568600/OsCPK20*	0.00%
Off-target 7	AGCTCTTCGACCGCATCGTCGCG	*Os01g0832300/OsCPK3*	23.91%
Off-target 8	AGCTCTTCGACCGCATCATCGCC	*Os01g0622600/OsCPK1*	6.52%

b

OsCPK3*-targeting sgRNA 2 / off-target site 2: *OsCPK21

5′-GUCGAUGGCCUUGAGCGGCG-3′

5′-GAA**GTCGATGACCTTGAGCGGCGAGT**CCTCCGATT-3′ WT

#30 5′-GAAGTCGATGACCTTGAGCGGCGAGTCCTCCGATT-3′ WT

5′-GAAGTCGATGACC-----CGGCGAGTCCTCCGATT-3′ -5

#46 5′-GAAGTCGATGACCTTGAGCGGCGAGTCCTCCGATT-3′ WT

5′-GAAGTCGATGACCTTGAGCG-CGAGTCCTCCGATT-3′ -1

OsCPK3*-targeting sgRNA 2 / off-target site 6: *OsCPK25

3′-CGCCGCUCAAGGCCAUCGAG-5′

5′-GACG**ACGCGCCGCTCAAGGCCACCGA**CTTCGGCCT-3′ WT

#34 5′-GACGACGCGCCGCTCAAGGCCACCGACTTCGGCCT-3′ WT

5′-GACGACGCG----TCAAGGCCACCGACTTCGGCCT-3′ -4

c

OsCPK28*-targeting sgRNA 2 / off-target site 2: *OsCPK29

3′-AGAAGCUGGCGUAGCAGCGG-5′

5′-GGCG**AGCTCTTCGACCGCATCGTCGCC**CGCGGCCA-3′ WT

#15 5′-GGCGAGCTCTTCGACCGCATCGTCGCCCGCGGCCA-3′ WT

5′-GGCGAGCT--------GCATCGTCGCCCGCGGCCA-3′ -8

#22 5′-GGCGAGCTCTT-----GCATCGTCGCCCGCGGCCA-3′ -5

5′-GGCG-----------CGCATCGTCGCCCGCGGCCA-3′ -11

OsCPK28*-targeting sgRNA 2 / off-target site 7: *OsCPK3

3′-AGAAGCUGGCGUAGCAGCGG-5′

5′-GGGG**AGCTCTTCGACCGCATCGTCGCG**CGGGGCCA-3′ WT

#22 5′-GGGGAGCTCTTCGACCGCATCGTCGCGCGGGGCCA-3′ WT

5′-GGGGAGCTCT-----CGCATCGTCGCGCGGGGCCA-3′ -5

#27 5′-GGGGAGCTCTTCGACCGCATCGTCGCGCGGGGCCA-3′ WT

5′-GGGGAGCTCT-CGACCGCATCGTCGCGCGGGGCCA-3′ -1

#38 5′-GGGGAGCTCTTCGACCGCATCGTCGCGCGGGGCCA-3′ WT

5′-GGGGAGCTCTtTCGACCGCATCGTCGCGCGGGGCCA-3′ +1

#44 5′-GGGGAGCTCTTCGACCGCATCGTCGCGCGGGGCCA-3′ WT

5′-GGGGAGCTCTT--ACCGCATCGTCGCGCGGGGCCA-3′ -2

OsCPK28*-targeting sgRNA 2 / off-target site 8: *OsCPK1

3′-AGAAGCUGGCGUAGCAGCGG-5′

5′-GGGG**AGCTCTTCGACCGCATCATCGCC**AGGGGGCA-3′ WT

#13 5′-GGGGAGCTCTTCGACCGCATCATCGCCAGGGGGCA-3′ WT

5′-GGGGAGCTC------CGCATCATCGCCAGGGGGCA-3′ -6

#35 5′-GGGGAGCTCTTCGACCGCATCATCGCCAGGGGGCA-3′ WT

5′-GGGGAGCTCT---ACCGCATCATCGCCAGGGGGCA-3′ -3

Figure S18. Off-target analysis of SpRY nuclease. **a** Summary of mutation frequencies in the potential off-target sites in the rice genome for SpRY nuclease in this study. The PAM sequences and the mismatches in the sgRNA sequences are highlighted in green and red, respectively. **b** Representative mutant alleles of off-targets detected in independent transgenic lines. WT, wild type; The PAM sequences, mismatches and target sequences are highlighted in green, red and bold, respectively; nucleotide deletions and insertions are indicated by dashes and lowercase letters, respectively.

Table S1. The complete nucleotide sequences of the rice codon-optimized *SpG*, *SpRY* and *sgRNA* fragments.

SpG-fg1	AAGCTTGGATCCGATCCAAATCCGAATCCGGCCATGGACTATAAGGATCACGATGGCGACTACAAGGATCATGACATTGACTATAAG<br>GATGACGACGATAAGATGGCACCTAAGAAGAAAAGGAAAGTCGGCATTCATGGCGTTCCGGCAGCCGACAAAAAGTATAGCATCGGC<br>CTCGATATTGGGACAAACTCTGTGGGCTGGGCGGTAATTACCGACGAGTACAAGGTGCCTAGTAAGAAATTTAAAGTGCTCGGAAAC<br>ACTGACAGGCACTCTATAAAGAAGAACCTGATCGGGGCACTGCTTTTCGACTCCGGAGAGACGGCGGAGGCGACGCGTCTCAAGCGT<br>ACCGCGCGCCGCAGGTACACAAGAAGGAAGAATAGGATCTGCTACTTGCAGGAAATCTTCAGTAACGAGATGGCGAAGGTCGACGAT<br>AGTTTCTTTCATCGGTTGGAAGAATCGTTCCTCGTAGAGGAGGACAAAAAGCACGAGCGTCACCCAATATTCGGGAATATTGTTGAC<br>GAGGTTGCCTACCATGAGAAATATCCTACAATATATCACCTCCGTAAGAAGCTTGTCGATTCAACTGATAAGGCTGATCTCAGACTC<br>ATCTATCTTGCCCTCGCACATATGATTAAGTTTCGTGGCCACTTCTTGATTGAAGGCGACCTCAACCCGGACAACTCAGATGTTGAC<br>AAGCTTTTTATACAGCTCGTCCAGACATATAACCAGCTGTTTGAAGAGAATCCCATCAATGCGAGTGGGGTTGATGCTAAAGCCATT<br>TTGTCCGCCAGGTTGTCCAAATCTCGCAGACTGGAAAACCTGATCGCACAGCTTCCCGGTGAAAAGAAAAACGGGCTCTTCGGCAAT<br>CTCATCGCACTGTCCCTCGGCCTCACCCCAAACTTCAAGTCTAACTTCGACCTGGCCGAGGATGCGAAGCTCCAGCTGTCAAAAGAT<br>ACATACGACGACGATTTGGACAATCTGCTTGCGCAAATAGGCGACCAGTATGCGGACCTGTTCCTGGCTGCCAAAAATCTGTCAGAT<br>GCAATCCTCCTGTCCGATATATTGCGTGTGAACACCGAAATCACGAAGGCACCGCTTAGCGCATCCATGATCAAGAGATACGACGAG<br>CACCATCAGGACCTCACACTCCTCAAGGCGCTTGTTCGTCAGCAGCTTCCCGAGAAATATAAGGAAATTTTTTTCGATCAAAGCAAG<br>AATGGATATGCTGGCTATATTGACGGTGGCGCTTCGCAGGAGGAGTTCTATAAATTCATTAAGCCGATTCTGGAGAAGATGGACGGA<br>ACGGAGGAGCTCCTCGTCAAGCTTAACCGGGAAGACCTGTTGCGGAAGCAGAGGACTTTTGATAACGGCTCTATTCCGCACCAAATC<br>CATCTGGGTGAGTTGCACGCAATCTTGAGAAGACAAGAGGATTTCTACCCGTTCCTTAAGGATAACAGAGAGAAGATAGAAAAAATA<br>CTGACCTTCAGGATACCATACTATGTGGGCCCACTGGCGCGCGGAAATAGTCGTTTCGCATGGATGACTAGAAAGTCCGAAGAAACG<br>ATCACGCCATGGAATTTTGAGGAAGTGGTCGACAAGGGCGCCTCTGCCCAGAGCTTCATCGAAAGGATGACCAATTTTGACAAAAAT<br>CTGCCTAACGAAAAGGTGCTTCCGAAGCACAGCCTGTTGTATGAATACTTCACAGTTTATAACGAGCTCACTAAGGTCAAGTACGTC<br>ACGGAGGGCATGCGTAAGCCTGCTTTCCTGTCTGGTGAACAAAAAAAGGCGATTGTGGACCTCCTTTTCAAGACGAACCGTAAAGTT<br>ACTGTGAAGCAACTGAAAGAGGATTACTTTAAGAAAATTGAGTGCTTCGACAGTGTGGAGATTTCCGGTGTCGAGGACCGGTTTAAC<br>GCCAGCCTGGGTACGTATCATGACCTGCTTAAAATTATCAAGGATAAAGATTTCCTGGATAATGAAGAGAACGAAGATATACTGGAG<br>GACATTGTGTTGACTTTGACCCTCTTCGAGGACAGAGAGATGATTGAGGAAAGACTGAAGACCTACGCACACCTTTTTGATGACAAG<br>GTCATGAAACAACTCAAGCGCCGGCGCTATACTGGCTGGGGCCGGCTTTCTCGCAAGCTCATCAATGGGATTCGGGATAAGCAATCA

	GGCAAGACAATTTTGGACTTCCTCAAATCCGACGGATTCGCAAATAGGAATTTTATGCAGCTGATACATGACGACTCTTTGACATTC<br>AAAGAAGACATACAGAAGGCTCAGGTCTCCGGCCAAGGAGATTCTTTGCACGAGCATATCGCTAACTTGGCAGGTAGCCCCGCCATA<br>AAAAAGGGCATTCTTCAAACGGTAAAAGTTGTTGACGAACTCGTGAAGGTTATGGGCCGTCATAAGCCGGAAAACATTGTTATTGAA<br>ATGGCTAGGGAAAATCAGACGACCCAGAAGGGACAGAAAAATAGCAGGGAGCGGATGAAGAGAATTGAAGAGGGAATTAAGGAGCTT<br>GGATCTCAGATTCTTAAGGAGCACCCTGTGGAGAACACCCAACTTCAGAATGAAAAGCTCTACCTTTACTACCTTCAAAACGGCCGG<br>GATATGTACGTCGATCAGGAACTTGACATTAACCGGTTGAGCGATTATGACGTTGACCATATTGTGCCCCAATCTTTCCTTAAAGAC<br>GACTCTATCGACAATAAAGTGCTGACGCGCAGCGATAAAAATCGCGGTAAGTCGGATAATGTCCCGTCGGAAGAGGTGGTTAAAAAA<br>ATGAAGAACTATTGGAGGCAACTCCTGAATGCCAAGCTGATCACTCAGAGGAAATTCGACAATCTCACCAAGGCAGAAAGGGGTGGA<br>CTTAGCGAGCTCGACAAGGCCGGTTTTATCAAAAGACAGCTGGTGGAGACACGCCAAATCACCAAACACGTTGCCCAGATCCTGGAT<br>TCGAGGATGAACACGAAGTATGACGAGAACGACAAGTTGATTAGGGAAGTCAAGGTCATCACTTTGAAGTCCAAGCTGGTGAGCGAC<br>TTTCGCAAAGACTTCCAGTTTTACAAAGTCAGGGAAATTAATAACTACCACCACGCCCACGACGCCTACCTTAACGCCGTGGTTGGC<br>ACAGCACTCATCAAGAAATACCCTAAGCTCGAATCTGAGTTCGTCTATGGCGACTATAAGGTCTACGACGTTAGAAAAATGATCGCG<br>AAATCTGAGCAGGAAATAGGCAAGGCAACTGCCAAGTACTTCTTCTATTCCAATATCATGAACTTTTTAAGACGGAGATTACCCTG<br>GCGAATGGTGAGATCCGCAAGCGCCCTTTGATTGAGA
SpG-fg2	AATGGTGAGATCCGCAAGCGCCCTTTGATTGAGACAAACGGAGAAACAGGAGAGATCGTATGGGACAAAGGGCGGGACTTTGCTACT<br>GTTAGGAAGGTGCTCTCTATGCCACAAGTTAACATTGTCAAAAAAACTGAAGTGCAGACAGGTGGGTTTAGCAAGGAATCTATCCTG<br>CCGAAGAGGAACTCTGACAAGCTGATCGCCCGCAAGAAAGATTGGGAcCCGAAAAAGTACGGAGGATTCTTGTGGCCCACAGTTGCG<br>TACTCCGTGCTTGTCGTGGCCAAAGTGGAGAAGGGCAAGTCTAAGAAGCTCAAGAGCGTCAAAGAGTTGTTGGGGATCACGATTATG<br>GAGCGGTCGTCTTTCGAAAAGAATCCGATAGATTTTCTCGAGGCCAAGGGTTATAAAGAAGTCAAGAAGGATCTTATCATCAAGCTC<br>CCTAAGTACTCCCTCTTTGAGCTTGAAAACGGACGGAAAAGAATGCTGGCTTCAGCGAAGCAGCTTCAGAAGGGTAATGAACTCGCT<br>CTGCCCTCAAAATATGTGAATTTCCTTTACCTGGCATCACACTATGAGAAGCTTAAGGGGTCTCCAGAGGACAACGAGCAGAAGCAA<br>CTGTTCGTTGAACAACACAAGCACTACCTTGACGAGATTATCGAGCAAATCAGCGAGTTTAGCAAGCGCGTTATACTGGCAGACGCA<br>AATCTTGATAAGGTCCTTAGCGCCTACAACAAGCATAGAGACAAACCCATCCGGGAGCAGGCCGAGAACATTATTCATCTCTTCACC<br>TTGACGAATCTTGGGGCCCCGGCCGCGTTCAAGTACTTCGATACTACCATAGACAGAAAGCAATATCGGTCGACAAAGGAAGTTCTT<br>GACGCCACGCTGATCCACCAAAGTATAACAGGCCTCTATGAGACACGCATCGACCTTTCGCAGTTGGGCGGTGACCGCCCCAAAAAG<br>AAGAGGAAAGTTGGCGGGTGAACTAGTGAATTC

SpRY-fg2	AATGGTGAGATCCGCAAGCGCCCTTTGATTGAGACAAACGGAGAAACAGGAGAGATCGTATGGGACAAAGGGCGGGACTTTGCTACT<br>GTTAGGAAGGTGCTCTCTATGCCACAAGTTAACATTGTCAAAAAAACTGAAGTGCAGACAGGTGGGTTTAGCAAGGAATCTATCAGG<br>CCGAAGAGGAACTCTGACAAGCTGATCGCCCGCAAGAAAGATTGGGAcCCGAAAAAGTACGGAGGATTCTTGTGGCCCACAGTTGCG<br>TACTCCGTGCTTGTCGTGGCCAAAGTGGAGAAGGGCAAGTCTAAGAAGCTCAAGAGCGTCAAAGAGTTGTTGGGGATCACGATTATG<br>GAGCGGTCGTCTTTCGAAAAGAATCCGATAGATTTTCTCGAGGCCAAGGGTTATAAAGAAGTCAAGAAGGATCTTATCATCAAGCTC<br>CCTAAGTACTCCCTCTTTGAGCTTGAAAACGGACGGAAAAGAATGCTGGCTTCAGCGAAGCAGCTTCAGAAGGGTAATGAACTCGCT<br>CTGCCCTCAAAATATGTGAATTTCCTTTACCTGGCATCACACTATGAGAAGCTTAAGGGGTCTCCAGAGGACAACGAGCAGAAGCAA<br>CTGTTCGTTGAACAACACAAGCACTACCTTGACGAGATTATCGAGCAAATCAGCGAGTTTAGCAAGCGCGTTATACTGGCAGACGCA<br>AATCTTGATAAGGTCCTTAGCGCCTACAACAAGCATAGAGACAAACCCATCCGGGAGCAGGCCGAGAACATTATTCATCTCTTCACC<br>TTGACGAGGCTTGGGGCCCCGAGAGCGTTCAAGTACTTCGATACTACCATAGACCCAAAGCAATATCGGTCGACAAAGGAAGTTCTT<br>GACGCCACGCTGATCCACCAAAGTATAACAGGCCTCTATGAGACACGCATCGACCTTTCGCAGTTGGGCGGTGACCGCCCCAAAAAG<br>AAGAGGAAAGTTGGCGGGTGAACTAGTGAATTC
GCCC-<br>type<br>*sgRNA*	GTCAGCGATGAGTACAGCAAGCCCCAGAGCTAGAAATAGCAAGTTGGGGTAAGGCTAGTCCGTTATCAACTTGAAAAAGTGGCACCG<br>AGTCGGTGCTTTTTTTTTGAGATTTCCAACCAGGTCCCTGGAGCCCATAGtctagtAACGGCCGCCAGTGTGCTGGAATTGCCCTTGG<br>ATCATGAACCAACGGCCTGGCTGTATTTGGTGGTTGTGTAGGGAGATGGGGAGAAGAAAAGCCCGATTCTCTTCGCTGTGATGGGCT<br>GGATGCATGCGGGGGAGCGGGAGGCCCAAGTACGTGCACGGTGAGCGGCCCACAGGGCGAGTGTGAGCGCGAGAGGCGGGAGGAACA<br>GTTTAGTACCACATTGCCCAGCTAACTCGAACGCGACCAACTTATAAACCCGCGCGCTGTCGCTTGTGTAGAGACCAAAGGAGGTCT<br>CAGCCCCAGAGCTAGAAATAGCAAGTTGGGGTAAGGCTAGTCCGTTATCAACTTGAAAAAGTGGCACCGAGTCGGTGCTTTTTTTGT<br>CCCTTCGAAGGGCAATTC
TadA8e	ATGTCAGAAGTCGAGTTCTCCCATGAGTATTGGATGAGGCACGCCCTCACTCTTGCGAAGAGGGCCAGGGACGAGAGGGAGGTGCCG<br>GTCGGTGCTGTCCTGGTCTTGAATAACAGGGTGATAGGCGAAGGTTGGAACAGGGCTATTGGCCTTCATGACCCTACTGCTCATGCG<br>GAAATCATGGCACTTAGACAGGGGGGCCTCGTTATGCAAAATTACCGCCTGATCGACGCCACTCTTTATGTCACATTTGAACCATGT<br>GTTATGTGTGCGGGCGCTATGATCCATTCACGCATAGGTCGCGTGGTTTTTGGAGTTCGCAACAGTAAACGTGGGGCTGCAGGCTCT<br>CTGATGAACGTTTTGAATTATCCGGGAATGAACCATAGAGTCGAAATCACAGAAGGGATTTTGGCAGACGAATGCGCGGCTCTTCTT<br>TGTGATTTTTACAGAATGCCCCGCCAAGTGTTTAATGCTCAAAAGAAAGCGCAGAGTAGCATCAACTCGGGGGGATCTTCTGGGGGC<br>TCGTCTGGTTCCGAGACTCCCGGAACTTCCGAGTCGGCAACACCTGAATCCTCCGGCGGCTCTTCGGGCGGATCTGAC

	AAAAAATACTCAATTGGTCTGGCTATTGGGACAAACTCTG

Table S2. List of oligonucleotides in this study.

Primer name	Primer sequence (5' - 3')	Used for
SpG-F3	GCGAATGCATCTAGATATCGGATCCGATCCAAATCCGAA	Assembling the full-length *SpG* and *SpRY* genes through overlapping-extension PCR-based method
SpG-R3	CTCTCCTGTTTCTCCGTTTGTCTCAATCAAAGGGCGCTTG	
SpG-F1a	CAAGCGCCCTTTGATTGAGACAAACGGAGAAACAGGAG	
SpG-R2	TTCACTAGTTCACCCGCCAAC	
SpRY-F1	AGGACGCGTCTCAAGCGTAC	Introducing A61R point mutation into SpG-fg1
SpRY-R1	CTCCGCCGTCTCTCCGGAGT	
UGI-F1	TCCGGCGGAAGTACAAAC	Fusing *hAID*Δ* and *UGI* to *SpRY* at the 5'and 3' ends, resulting in *rBE66*
rAPO-R1	AGCAAGTCCGATTGAATACT	
OsCas9-Fg1-F1	ATTGGGACAAACTCTGTGG	
OsCas9-Fg2-R1	GTCACCGCCCAACTGCGA	
TadA8e-F1	CTTGGATCCATGTCAGAAGTCGAGTTC	Fusing *TadA8e* to the 5' end of *SpRY*, resulting in *rBE62*
TadA8e-F2	CAGAGTTTGTCCCAATAGCC	
SpRY-F2	GGCTATTGGGACAAACTCTG	
SpRY-R2	TTCACTAGTTCACCCGCCAACTTTC	
gRNA4-NG-F1	GTCCCTTCGAAGGGCAATTC	Generating pENTR4:sgRNA4-NG construct
gRNA4-NG-R1	CTTGCTGTACTCATCGCTGAC	

gRNA4-NG-F2	GTCAGCGATGAGTACAGCAAG	
gRNA4-NG-R2	GAATTGCCCTTCGAAGGGAC	
gOsCERK1-F3a	TGTTGGCCTTCCTTGGGATCCGG	Knocking out the endogenous *OsCERK1* gene with an NGA PAM
gOsCERK1-R3a	AAACCCGGATCCCAAGGAAGGCC	
OsCERK1-F3	GACGTCTACGCCTTTGGTGT	Detecting nucleotide changes of *OsCERK1*
OsCERK1-R3	GTCAGCTGCAAAATGCAATG	
gOsRLCK185-F1a	GTGTGCGTGCCTCAATTTGTGCAC	Knocking out the endogenous *OsRLCK185* gene with an NGC PAM
gOsRLCK185-R1a	AAACGTGCACAAATTGAGGCACG	
OsRLCK185-F2	TCCATGGCCTTGTTCCTCTT	Detecting nucleotide changes of *OsRLCK185*
OsRLCK185-R2	TGCTGCTAGACACATCCACA	
gOsGSK4-FGG	GTGTGTTGTTGGAACTGGATCCTT	Knocking out the endogenous *OsGSK4* gene with an NGG PAM
gOsGSK4-RGG	AAACAAGGATCCAGTTCCAACAAC	
OsGSK4-F1	GGAAATGATCCAGTCACAGGT	Detecting nucleotide changes of *OsGSK4*
OsGSK4-R1	TCGGGAACAAACTCCATGAC	
gOsPAL5-F2	TGTTGGAGGAATTCAGGATCCTGA	Knocking out the endogenous *OsPAL5* gene with an NGG PAM
gOsPAL5-R2	AAACTCAGGATCCTGAATTCCTCC	
OsPAL5-F1	AACAACGGAATCAGAAAGCTG	Detecting nucleotide changes of *OsPAL5*

OsPAL5-R1	CACCGGAGAGAAGCTCAAGT	
gOsGSK4-FGT	TGTTGATACAACACCAAAGGATCC	Knocking out the endogenous *OsGSK4* gene with an NGT PAM
gOsGSK4-RGT	AAACGGATCCTTTGGTGTTGTATC	
OsGSK4-F1	GGAAATGATCCAGTCACAGGT	Detecting nucleotide changes of *OsGSK4*
OsGSK4-R1	TCGGGAACAAACTCCATGAC	
gOsETR2-FGT	GTGTGTCCCATCTCTGTGGATCCG	Knocking out the endogenous *OsETR2* gene with an NGT PAM
gOsETR2-RGT	AAACCGGATCCACAGAGATGGGAC	
OsETR2-F1	TGGTTCGTTCGTTTGTTTGA	Detecting nucleotide changes of *OsETR2*
OsETR2-R1	ATGGTGATGAGGTGGGTGAG	
gOsCERK1-FGC	TGTTGAAGGCCTTCCTTGGGATCC	Knocking out the endogenous *OsCERK1* gene with an NGC PAM
gOsCERK1-RGC	AAACGGATCCCAAGGAAGGCCTTC	
OsCERK1-F3	GACGTCTACGCCTTTGGTGT	Detecting nucleotide changes of *OsCERK1*
OsCERK1-R3	GTCAGCTGCAAAATGCAATG	
gOsMPK8-F2	TGTTGCGCCAGAGATGGATTTCTT	Knocking out the endogenous *OsMPK8* gene using the GTTT-type sgRNA scaffold
gOsMPK8-R2	AAACAAGAAATCCATCTCTGGCGC	
gOsMPK8-F3	GTGTGCTATTACGGCATCCTGATA	Knocking out the endogenous *OsMPK8* gene using the GTTT-type sgRNA scaffold
gOsMPK8-R3	AAACTATCAGGATGCCGTAATAGC	

OsMPK8-F	CTTTGCATCAGAAGGGCAGG	Detecting nucleotide changes of *OsMPK8*
OsMPK8-R	CAGTCATCAATAAACGCTGTGCT	
gOsMPK9-F3	TGTTGAGAGGAGCACGAGAGAGAG	Knocking out the endogenous *OsMPK9* gene using the GTTT-type sgRNA scaffold
gOsMPK9-R3	AAACCTCTCTCTCGTGCTCCTCTC	
gOsMPK9-F4	GTGTGCCTATGCTTATCCGAACAG	Knocking out the endogenous *OsMPK9* gene using the GTTT-type sgRNA scaffold
gOsMPK9-R4	AAACCTGTTCGGATAAGCATAGGC	
OsMPK9-F3	TTTACGGCACCACGAATCCA	Detecting nucleotide changes of *OsMPK9*
OsMPK9-R3	AACATCAACGGGCACCCATA	
OsMPK9-F4	CCAGGTGCACTTGTGTTTCA	
OsMPK9-R4	TGTTAGTACCAACGCCTGCC	
gOsMPK10-F2	TGTTGACACTGGAGAGAAAGTGGC	Knocking out the endogenous *OsMPK10* gene using the GTTT-type sgRNA scaffold
gOsMPK10-R2	AAACGCCACTTTCTCTCCAGTGTC	
gOsMPK10-F3	GTGTGTGACTGATGTTGCTCTGGA	Knocking out the endogenous *OsMPK10* gene using the GTTT-type sgRNA scaffold
gOsMPK10-R3	AAACTCCAGAGCAACATCAGTCAC	
gOsMPK10-F2	TGTTGACACTGGAGAGAAAGTGGC	Knocking out the endogenous *OsMPK10* gene using the GCCC-type sgRNA scaffold
gOsMPK10-NG-R2	GGGCGCCACTTTCTCTCCAGTGTC	
gOsMPK10-F3	GTGTGTGACTGATGTTGCTCTGGA	Knocking out the endogenous *OsMPK10* gene

gOsMPK10-NG-R3	GGGCTCCAGAGCAACATCAGTCAC	using the GCCC-type sgRNA scaffold
OsMPK10-F2	TGATTGTGAGGACAGACGGT	Detecting nucleotide changes of *OsMPK10*
OsMPK10-R2	GTGACAGTTCCCTACCAGTGT	
OsMPK10-F3	CCAACAGGTGCTTTGTGGTTAAT	
OsMPK10-R3	AGGGAACAACACCGACCTTT	
gOsCPK1-F1	TGTTGACTCGGTCAACCTCATCAT	Knocking out the endogenous *OsCPK1* gene using the GTTT-type sgRNA scaffold
gOsCPK1-R1	AAACATGATGAGGTTGACCGAGTC	
gOsCPK1-F2	GTGTGAGACCGAAGTCAGTGGCCT	Knocking out the endogenous *OsCPK1* gene using the GTTT-type sgRNA scaffold
gOsCPK1-R2	AAACAGGCCACTGACTTCGGTCTC	
OsCPK1-F	GTCACCTACCTCGTCACCCA	Detecting nucleotide changes of *OsCPK1*
OsCPK1-R	TTTCAGACCTGTCAAACCCA	
gOsCPK2-F1	TGTTGAGGACGTCCGGAGCATCTA	Knocking out the endogenous *OsCPK2* gene using the GTTT-type sgRNA scaffold
gOsCPK2-R1	AAACTAGATGCTCCGGACGTCCTC	
gOsCPK2-F2	GTGTGGCTTGAGGTCGCGGTGGAT	Knocking out the endogenous *OsCPK2* gene using the GTTT-type sgRNA scaffold
gOsCPK2-R2	AAACATCCACCGCGACCTCAAGCC	
OsCPK2-F	GGCAACTGCTGTCCTGGCTCT	Detecting nucleotide changes of *OsCPK2*
OsCPK2-R	ATGGCGGCAACGGATGAA	

gOsCPK3-F1	TGTTGGGGAAGTGAGACGACTTGA	Knocking out the endogenous *OsCPK3* gene using the GTTT-type sgRNA scaffold
gOsCPK3-R1	AAACTCAAGTCGTCTCACTTCCCC	
gOsCPK3-F2	GTGTGTCGATGGCCTTGAGCGGCG	Knocking out the endogenous *OsCPK3* gene using the GTTT-type sgRNA scaffold
gOsCPK3-R2	AAACCGCCGCTCAAGGCCATCGAC	
gOsCPK3-F1	TGTTGGGGAAGTGAGACGACTTGA	Knocking out the endogenous *OsCPK3* gene using the GCCC-type sgRNA scaffold
gOsCPK3-NG-R1	GGGCTCAAGTCGTCTCACTTCCCC	
gOsCPK3-F2	GTGTGTCGATGGCCTTGAGCGGCG	Knocking out the endogenous *OsCPK3* gene using the GCCC-type sgRNA scaffold
gOsCPK3-NG-R2	GGGCCGCCGCTCAAGGCCATCGAC	
OsCPK3-F1	CAATTCCTTCTCCTCCCATC	Detecting nucleotide changes of *OsCPK3*
OsCPK3-R1	GTACGTCACCCCGAACTCCC	
OsCPK3-F2	GCAAGTCCATCTCCAAGCGG	
OsCPK3-R2	GTATTCCACCCAAAACAGACCA	
gOsCPK4-F1	TGTTGTCTCATCCCACACTGCGAC	Knocking out the endogenous *OsCPK4* gene using the GTTT-type sgRNA scaffold
gOsCPK4-R1	AAACGTCGCAGTGTGGGATGAGAC	
gOsCPK4-F2	GTGTGCCGTCAAGCGCATCGACAA	Knocking out the endogenous *OsCPK4* gene using the GTTT-type sgRNA scaffold
gOsCPK4-R2	AAACTTGTCGATGCGCTTGACGGC	
gOsCPK4-F1	TGTTGTCTCATCCCACACTGCGAC	Knocking out the endogenous *OsCPK4* gene using

gOsCPK4-NG-R1	GGGCGTCGCAGTGTGGGATGAGAC	the GCCC-type sgRNA scaffold
gOsCPK4-F2	GTGTGCCGTCAAGCGCATCGACAA	Knocking out the endogenous *OsCPK4* gene using the GCCC-type sgRNA scaffold
gOsCPK4-NG-R2	GGGCTTGTCGATGCGCTTGACGGC	
OsCPK4-F	GACCAAACAACCTCCCTC	Detecting nucleotide changes of *OsCPK4*
OsCPK4-R	TTCGCACTAGCTCAATCC	
gOsCPK5-F3	TGTTGAGTACTTCGCCAGCTTCCG	Knocking out the endogenous *OsCPK5* gene using the GTTT-type sgRNA scaffold
gOsCPK5-R3	AAACCGGAAGCTGGCGAAGTACTC	
gOsCPK5-F4	GTGTGGAGCGCGTAGTGCTCGCTC	Knocking out the endogenous *OsCPK5* gene using the GTTT-type sgRNA scaffold
gOsCPK5-R4	AAACGAGCGAGCACTACGCGCTCC	
OsCPK5-F	TCCAACTCCCTCCATTGCTC	Detecting nucleotide changes of *OsCPK5*
OsCPK5-R	ATGTGAACGTACTGCGGGTC	
gOsCPK8-F4	TGTTGCGAACCCCTTCACCGTCGC	Knocking out the endogenous *OsCPK8* gene using the GTTT-type sgRNA scaffold
gOsCPK8-R4	AAACGCGACGGTGAAGGGGTTCGC	
gOsCPK8-F5	GTGTGTCGATATCGACTTGCACGC	Knocking out the endogenous *OsCPK8* gene using the GTTT-type sgRNA scaffold
gOsCPK8-R5	AAACGCGTGCAAGTCGATATCGAC	
OsCPK8-F	AACTCACACGGCGAAGCAC	Detecting nucleotide changes of *OsCPK8*
OsCPK8-R	ACAGTCCACACGGAGACAC	

gOsCPK14-F1	TGTTGGATCGACTTGACGTCCTCC	Knocking out the endogenous *OsCPK14* gene using the GTTT-type sgRNA scaffold
gOsCPK14-R1	AAACGGAGGACGTCAAGTCGATCC	
gOsCPK14-F2	GTGTGAGGCGGCGTGCTCGGTGTA	Knocking out the endogenous *OsCPK14* gene using the GTTT-type sgRNA scaffold
gOsCPK14-R2	AAACTACACCGAGCACGCCGCCTC	
gOsCPK14-F1	TGTTGGATCGACTTGACGTCCTCC	Knocking out the endogenous *OsCPK14* gene using the GCCC-type sgRNA scaffold
gOsCPK14-NG-R1	GGGCGGAGGACGTCAAGTCGATCC	
gOsCPK14-F2	GTGTGAGGCGGCGTGCTCGGTGTA	Knocking out the endogenous *OsCPK14* gene using the GCCC-type sgRNA scaffold
gOsCPK14-NG-R2	GGGCTACACCGAGCACGCCGCCTC	
OsCPK14-F	AACCGAACCCAAACCCGC	Detecting nucleotide changes of *OsCPK14*
OsCPK14-R	GAAGTCGGTGGCCTTGAGAG	
gOsCPK20-F1	TGTTGCGAGTACAACCGGTCGTCG	Knocking out the endogenous *OsCPK20* gene using the GTTT-type sgRNA scaffold
gOsCPK20-R1	AAACCGACGACCGGTTGTACTCGC	
gOsCPK20-F2	GTGTGATAAGCATGGAGTGATGCA	Knocking out the endogenous *OsCPK20* gene using the GTTT-type sgRNA scaffold
gOsCPK20-R2	AAACTGCATCACTCCATGCTTATC	
gOsCPK20-F1	TGTTGCGAGTACAACCGGTCGTCG	Knocking out the endogenous *OsCPK20* gene using the GCCC-type sgRNA scaffold
gOsCPK20-NG-R1	GGGCCGACGACCGGTTGTACTCGC	
gOsCPK20-F2	GTGTGATAAGCATGGAGTGATGCA	Knocking out the endogenous *OsCPK20* gene

gOsCPK20-NG-R2	GGGCTGCATCACTCCATGCTTATC	using the GCCC-type sgRNA scaffold
OsCPK20-F1	CTCTCGCCTCCTTCTCCT	Detecting nucleotide changes of *OsCPK20*
OsCPK20-R1	GGCATTGTCGTCCTCGTAGGTGT	
OsCPK20-F2	ATCGAGTTCACTGGTGGAGA	
OsCPK20-R2	TGACAACTGAGAACTGACTGAC	
gOsCPK21-F1	TGTTGATCAGCAAGATCTCCCTCG	Knocking out the endogenous *OsCPK21* gene using the GTTT-type sgRNA scaffold
gOsCPK21-R1	AAACCGAGGGAGATCTTGCTGATC	
gOsCPK21-F2	GTGTGTGTGCTTCCGGATCGTCTT	Knocking out the endogenous *OsCPK21* gene using the GTTT-type sgRNA scaffold
gOsCPK21-R2	AAACAAGACGATCCGGAAGCACAC	
OsCPK21-F	ACACACACAGAGGAGGAG	Detecting nucleotide changes of *OsCPK21*
OsCPK21-R	AATGGGAGAAGAAGAAGA	
gOsCPK27-F1	TGTTGGGAGGCCGTTCTTGGCGAA	Knocking out the endogenous *OsCPK27* gene using the GTTT-type sgRNA scaffold
gOsCPK27-R1	AAACTTCGCCAAGAACGGCCTCCC	
gOsCPK27-F2	GTGTGGCTTGATGTGCGCCGGCTT	Knocking out the endogenous *OsCPK27* gene using the GTTT-type sgRNA scaffold
gOsCPK27-R2	AAACAAGCCGGCGCACATCAAGCC	
OsCPK27-F	GAGCGGTGTTGTGTGAAG	Detecting nucleotide changes of *OsCPK27*
OsCPK27-R	CGGATGGAGATGATGTTG	

gOsCPK28-F1	TGTTGCGAGTACGCGTGCAAGTCC	Knocking out the endogenous *OsCPK28* gene using the GTTT-type sgRNA scaffold
gOsCPK28-R1	AAACGGACTTGCACGCGTACTCGC	
gOsCPK28-F2	GTGTGGCGACGATGCGGTCGAAGA	Knocking out the endogenous *OsCPK28* gene using the GTTT-type sgRNA scaffold
gOsCPK28-R2	AAACTCTTCGACCGCATCGTCGCC	
gOsCPK28-F1	TGTTGCGAGTACGCGTGCAAGTCC	Knocking out the endogenous *OsCPK28* gene using the GCCC-type sgRNA scaffold
gOsCPK28-NG-R1	GGGCGGACTTGCACGCGTACTCGC	
gOsCPK28-F2	GTGTGGCGACGATGCGGTCGAAGA	Knocking out the endogenous *OsCPK28* gene using the GCCC-type sgRNA scaffold
gOsCPK28-NG-R2	GGGCTCTTCGACCGCATCGTCGCC	
OsCPK28-F	GATCGGAAAATGCAGCCTGA	Detecting nucleotide changes of *OsCPK28*
OsCPK28-R	CAAAGTGCGCAGAATCCGAG	
gOsMPK3-F1	TGTTGCAAGCCCATCGGGCGAGGA	Knocking out the endogenous *OsMPK3* gene using the GTTT-type sgRNA scaffold
gOsMPK3-R1	AAACTCCTCGCCCGATGGGCTTGC	
gOsMPK3-F2	TGTTGCTGAAACTTCTCCGGCATC	Knocking out the endogenous *OsMPK3* gene using the GTTT-type sgRNA scaffold
gOsMPK3-R2	AAACGATGCCGGAGAAGTTTCAGC	
OsMPK3-F	ACCATAGCCGAGCAACTGAA	Detecting nucleotide changes of *OsMPK3*
OsMPK3-R	AAGCAATCATCAAGGGCCA	
gOsMPK4-F3	GTGTGTGACACCAAGTATGTGCCA	Knocking out the endogenous *OsMPK4* gene

gOsMPK4-R3	AAACTGGCACATACTTGGTGTCAC	using the GTTT-type sgRNA scaffold
gOsMPK4-F4	GTGTGCTCCTCTATAAACCGTGCA	Knocking out the endogenous *OsMPK4* gene using the GTTT-type sgRNA scaffold
gOsMPK4-R4	AAACTGCACGGTTTATAGAGGAGC	
OsMPK4-F	TGCATTTTCTCCCCTTCCTGATT	Detecting nucleotide changes of *OsMPK4*
OsMPK4-R	ATACGTCATGGTGGAACCGCT	
gOsCOI2-F4	GTGTGAGCGCGTCGACCCTGCACC	Base editing the endogenous *OsCOI2* gene with an NGT PAM
gOsCOI2-R4	AAACGGTGCAGGGTCGACGCGCTC	
gOsCOI2-F7	GTGTGTCGACCCTGCACCAGTGGC	Base editing the endogenous *OsCOI2* gene with an NGC PAM
gOsCOI2-R7	AAACGCCACTGGTGCAGGGTCGAC	
gOsCOI2-F8	GTGTGGAGCGCGTCGACCCTGCAC	Base editing the endogenous *OsCOI2* gene with an NAG PAM
gOsCOI2-R8	AAACGTGCAGGGTCGACGCGCTCC	
OsCOI2-F1	CAACTTCCGCTTTTTCCTTG	Detecting nucleotide changes of *OsCOI2*
OsCOI2-R1	TTGAACGAGGAGAGCATGTG	
gOsMPK13-F16	GTGTGGGACATGGAGTTCTTTACG	Base editing the endogenous *OsMPK13* gene with an NAA PAM
gOsMPK13-R16	AAACCGTAAAGAACTCCATGTCCC	
OsMPK13-F1	TGTGTGCCATTACAGTTTCCA	Detecting nucleotide changes of *OsMPK13*
OsMPK13-R1	CCTGAACTCCCTTCGGGTAG	

gBSRK1-F5	GTGTGTTCCTAGAACAAACTACAC	Gene correction of the defective endogenous *BSR-K1* gene with an NGA PAM
gBSRK1-R5	AAACGTGTAGTTTGTTCTAGGAAC	
gBSRK1-F6	GTGTGTCCTAGAACAAACTACACT	Gene correction of the defective endogenous *BSR-K1* gene with an NAG PAM
gBSRK1-R6	AAACAGTGTAGTTTGTTCTAGGAC	
Bsr-kit-F1	GTGAGATGCAAAGCTCGTTGG	Detecting nucleotide changes of *BSR-K1*
Bsr-kit-R1	CAGGGTGTTGAACAGTTCCG	
gBsrd1-F1	GTGTGTTTAACTGATAAATATAAG	Gene correction of the defective endogenous *BSR-D1* gene with an NAT PAM
gBsrd1-R1	AAACCTTATATTTATCAGTTAAAC	
Bsrd1-F	CATCCACCGTTCCACACGA	Detecting nucleotide changes of *BSR-D1*
Bsrd1-R	CAGCTCGATTTGCTCGCAC	
gOsGSK4-FAC	TGTTGTATAGCTAATAGTCTAGAG	Base editing the endogenous *OsGSK4* gene with an NAC PAM
gOsGSK4-RAC	AAACCTCTAGACTATTAGCTATAC	
OsGSK4-F1	GGAAATGATCCAGTCACAGGT	Detecting nucleotide changes of *OsGSK4*
OsGSK4-R1	TCGGGAACAAACTCCATGAC	
gMPK8-2 off-1-F2	AACCTGGTGGAGGTCTGTATC	Detecting the potential off-target mutation of *OsMPK8*-targeting sgRNA 1
gMPK8-2 off-1-R2	CAGCTGCTTAGCTCTTGGGG	
gMPK10-3 off-1-F1	TCTTAACAGTGGCACCAGGGA	Detecting the potential off-target mutation of

gMPK10-3 off-1-R1	CGGCAGCAACAATACCGTTAAG	*OsMPK10*-targeting sgRNA 2
gMPK10-3 off-2-F1	TATGCAATCCAGTCATCAAACTCA	Detecting the potential off-target mutation of *OsMPK10*-targeting sgRNA 2
gMPK10-3 off-2-R1	GCTCATGTGTGGGGTTTGTAG	
gMPK10-3 off-3-F1	AGGTTATGCGGCAGAAATGT	Detecting the potential off-target mutation of *OsMPK10*-targeting sgRNA 2
gMPK10-3 off-3-R1	TCAGAGGATTTCGAGTGCCG	
gCPK3-2 off-1-F1	GTTATGGCGAGCTAAGGTACG	Detecting the potential off-target mutation of *OsCPK3*-targeting sgRNA 2
gCPK3-2 off-1-R1	CAACATCGCGGAGTTCAGGG	
gCPK3-2 off-2-F2	ATATACCTCCCCAGAATGGAGGG	Detecting the potential off-target mutation of *OsCPK3*-targeting sgRNA 2
gCPK3-2 off-2-R2	CGAACACTACACATCATCTTCTTC	
OsCPK1-F	GTCACCTACCTCGTCACCCA	Detecting the potential off-target mutation of *OsCPK3*-targeting sgRNA 2
OsCPK1-R	TTTCAGACCTGTCAAACCCA	
gCPK3-2 off-4-F3	GAGCTGTTCGACCGGATCG	Detecting the potential off-target mutation of *OsCPK3*-targeting sgRNA 2
gCPK3-2 off-4-R3	GTTCAAGCGTGCATGCATTG	
gCPK3-2 off-5-F2	AAGCGGAAGCTACTCACCGA	Detecting the potential off-target mutation of *OsCPK3*-targeting sgRNA 2
gCPK3-2 off-5-R2	GCCTACCTACCTGCCCAGAA	
gCPK3-2 off-6-F1	CACCTCTCCGGCCAGCCA	Detecting the potential off-target mutation of *OsCPK3*-targeting sgRNA 2
gCPK3-2 off-6-R1	GTCCGCGAAGGATGGCGGT	

gCPK28-2 off-1-F1	CATGGAGAGGCCGAAATCAGT	Detecting the potential off-target mutation of *OsCPK28*-targeting sgRNA 1
gCPK28-2 off-1-R1	AGAAGCTTGGGTGTCCGTAG	
gCPK28-1 off-2-F1	CGAACACCACCCAGCAGCCT	Detecting the potential off-target mutation of *OsCPK28*-targeting sgRNA 1
gCPK28-1 off-2-R1	CGCTCCGTGTAGTGCCCCTT	
gCPK28-2 off-1-F1	CATGGAGAGGCCGAAATCAGT	Detecting the potential off-target mutation of *OsCPK28*-targeting sgRNA 2
gCPK28-2 off-1-R1	AGAAGCTTGGGTGTCCGTAG	
OsCPK29-F1	GCGTGCAAGTCGATCAGCAA	Detecting the potential off-target mutation of *OsCPK28*-targeting sgRNA 2
OsCPK29-R1	GCATGAGCATGTGGGTGGTG	
gCPK28-2 off-4-F1	GCCAAAATCGGTGGCTTTGA	Detecting the potential off-target mutation of *OsCPK28*-targeting sgRNA 2
gCPK28-2 off-4-R1a	GCTACGCCTGCAAGTCCATC	
gCPK28-2 off-5-F3	GTGAGTTCCTTCCTACCTGG	Detecting the potential off-target mutation of *OsCPK28*-targeting sgRNA 2
gCPK28-2 off-5-R3	GAGGTGGCAGTACAGGAGAT	
gCPK28-2 off-6-F1	TTCAAAGAGGCAGGCATGGA	Detecting the potential off-target mutation of *OsCPK28*-targeting sgRNA 2
gCPK28-2 off-6-R1	CTCCAAGAAGAAGCTCCGCA	
OsCPK3-F2	GCAAGTCCATCTCCAAGCGG	Detecting the potential off-target mutation of *OsCPK28*-targeting sgRNA 2
OsCPK3-R2	GTATTCCACCCAAAACAGACCA	
OsCPK1-F	GTCACCTACCTCGTCACCCA	Detecting the potential off-target mutation of

OsCPK1-R	TTTCAGACCTGTCAAACCCA	*OsCPK28*-targeting sgRNA 2

Table S3. Rice genes for targeted genome editing in this study.

Gene Name	Gene Identifier	Plasmid	Application
OsMPK3	Os02g0148100	pUbi:SpRY	Gene knockout
OsMPK4	Os06g0699400	pUbi:SpRY	Gene knockout
OsMPK8	Os01g0665200	pUbi:SpRY	Gene knockout
OsMPK9	Os05g0582400	pUbi:SpRY	Gene knockout
OsMPK10	Os01g0629900	pUbi:SpRY	Gene knockout
OsMPK13	Os02g0135200	pUbi:rBE62	Adenine base editing
OsCPK1	Os01g0622600	pUbi:SpRY	Gene knockout
OsCPK2	Os01g0808400	pUbi:SpRY	Gene knockout
OsCPK3	Os01g0832300	pUbi:SpRY	Gene knockout
OsCPK4	Os02g0126400	pUbi:SpRY	Gene knockout
OsCPK5	Os02g0685900	pUbi:SpRY	Gene knockout
OsCPK8	Os03g0808600	pUbi:SpRY	Gene knockout
OsCPK14	Os05g0491900	pUbi:SpRY	Gene knockout
OsCPK20	Os07g0568600	pUbi:SpRY	Gene knockout
OsCPK21	Os08g0540400	pUbi:SpRY	Gene knockout
OsCPK27	Os12g0486600	pUbi:SpRY	Gene knockout
OsCPK28	Os12g0169800	pUbi:SpRY	Gene knockout
OsCERK1	Os08g0538300	pUbi:SpG	Gene knockout
OsGSK4	Os06g0547900	pUbi:SpG	Gene knockout
OsGSK4	Os06g0547900	pUbi:rBE62	Adenine base editing
OsETR2	Os04g0169100	pUbi:SpG	Gene knockout
OsCOI2	Os03g0265500	pUbi:rBE66	Cytosine base editing
OsGS1	Os02g0735200	pUbi:rBE62	Adenine base editing
BSR-K1	Os10g0548200	pUbi:rBE66	Cytosine base editing

BSR-D1	Os03g0437200	pUbi:rBE62	Adenine base editing
