# Supplementary material for: SpRY greatly expands the genome editing scope in rice with highly flexible PAM recognition
Source: Genome Biol. 2021 Jan 4;22:6. doi: 10.1186/s13059-020-02231-9 (PMC7780387; doi:10.1186/s13059-020-02231-9)
Supplement: Supplementary file 2 — Additional file 2. Review history. [file 13059_2020_2231_MOESM2_ESM.docx]

Review History

**First round of review**

**Reviewer 1**

**Are you able to assess all statistics in the manuscript, including the appropriateness of statistical tests used?**

There are no statistics in the manuscript.

**Comments to author:**

In the manuscript of Xu et al., the authors thoroughly tested the gene editing performance of SpCas9 variants, SpG and SpRY, and SpRY-derived base editors in transgenic rice and obtained the following findings: (1) SpG performs well on NGD PAMs but is less efficient than SpCas9-NG; (2) SpRY performs well on a broader range of PAMs than SpG, exhibiting a preference to NGD and NAN PAMs; (3) both SpRY-derived cytosine and adenine base editors can efficiently induce C-to-T or A-to-G conversions in the target sites with non-canonical PAMs; (4) SpRY can induce considerable self-editing events on sgRNA transgenes, whereas SpRY-derived base editors induce much less self-editing events. Overall, this is a timely and well-designed research that will have a broad impact on the genome editing practice in plant research and crop engineering. This reviewer only has some minor comments:

1. Line 143, a conjunction is needed right before 11.11%.

2. Line 151, Figure 2a, "di-allelic" should be "bi-allelic"?

3. Line 153, the sentence "being the ratios ... relatively higher" should be reworded.

4. Lines 238-240, where to find the information about indel positions? Is there any reference to justify the reasoning that the indel can be induced by the deaminase?

5. Fig. 2, in some cases, the sgRNA-editing frequencies were extremely high, whereas the on-target editing frequencies were 0 or very low in the same transgenic rice. It is very likely that disruption of sgRNA sequences by self-editing has prevented on-target editing. The authors can highlight these data in Discussion between lines 302-305.

6. Fig. 3d and 3g, it is unusual that the base editing occurred in BSR-K1 skipped the two central C. Also, only C-to-G rather than C-to-T substitution occurred for the two outside C. It is worthy to mention this to readers.

**Reviewer 2**

**Are you able to assess all statistics in the manuscript, including the appropriateness of statistical tests used?**

There are no statistics in the manuscript.

**Comments to author:**

In this study, Xu et al. developed new plant genome editing tools using SpG and SpRY variants and showed that SpRY can expand the target scopes in genome editing of rice. They further described a novel cytosine and adenine base editors using the SpRY which can expand target selectivity for base editing. The authors also found that SpRY could target the sgRNA sequence in the T-DNA in transgenic plants. Overall, this study could provide useful tools for plant research and crop breeding. However, I have several concerns regarding the experimental design and results of the nuclease activity and PAM preference of SpRY.

1.

I could not understand how authors evaluate mutation efficiency of transgenic calli.

For example, in Fig 1, 25/40 (62.5%) may indicate mutations were detected in 25 calli out of 40 calli. Then, 11 calli out of 25 calli were shown to be mono-allelic mutation.

However, I think each transformed callus should be chimeric and mono-allelic and bi-allelic mutations could be occurred in different cells in the same clump of callus.

2.

For the targeted mutagenesis using SpRY, authors used two gRNAs per transformation construct. I'm afraid that this experimental design could not evaluate the editing activity and PAM preference of SpRY properly. Since, it has been reported that additional catalytically dead Cas9 binding at proximal locations improves Cas9 activity (Chen et al., Nature Communications, 2017; Liu et al., Genome Biology, 2019). As shown in Fig. 2, mutations were introduced into only one of the two targets in several genes. Mutation efficiencies could be affected by Cas9 bound to the other target site. Thus, it is inappropriate to conclude nuclease activity and PAM preference of SpRY on the basis of these mutation efficiencies. Authors have to show the mutation efficiency using a single sgRNA per transformation construct.

3.

The data in this manuscript showing the application of base editors using SpRY is very limited, especially cytosine base editors were evaluated with only NGC and NAG PAMs. target sites. These experiments are insufficient to demonstrate the superiority of SpRY. The adenine base editors' experiments showed base substitutions in the NAA and NAT PAM target sites, demonstrating the advantages of using SpRY compared to the cytosine base editors' experiments. However, as described by the authors, the mutation efficiencies by SpRY greatly depend on the target locus. Therefore, more targets must be tested for each PAM and examined for base substitution rates.

4.

The authors found that SpRY not only targeted genome but also self-targeted sgRNA sequence in T-DNA in transgenic plants. However, according to the previous result of a high-throughput PAM determination assay (Walton et al., 2020, Science), SpRY seems to rarely recognize GTTT as a PAM. Therefore, it would be better to show in vitro cleave assay at GTTT PAM site, if possible.

Other comments:

Line 59

"double-stranded break (DSB)" should be "DNA double-strand break (DSB)".

Line 63 - 68

I guess the description of the base editor in the background should be a bit more detailed.

Line 279 - 280

Sretenovic and colleagues have already reported cytosine base editors and adenine base editors using iSpyMacCas9 which can recognize NAAR PAMs in rice (https://doi.org/10.1016/j.xplc.2020.100101). Hence, the authors' description "this is the first time a base editor which recognized non-G PAM sites in plants is described" is incorrect.

Line 335 - 336

As mentioned above, the number of targets examined in this manuscript is not sufficient to prove efficient base editing at minimal NA PAM site.

Line 367

"adenine" should be deleted.

Figure 3c

The sequence information appears to be wrong. The position of PAM sequence shown in the pale purple arrows is off, and target gene and base editor names and rBE66 are deleted.

Figure 3d

The sequence is opposite to the other figures (3g, 3h) and is not easy to see.

Figure 4f

Target region should be mentioned like Figure 3h.

Figure S1 - S18

These supplementary figures are fully incomplete.

(1)	There are too many errors in target sequence information, such as Figure S1a (no-bold characters), S2c (misalignment of position).

(2)	In supplementary figures, the authors showed sgRNA sequences, but only Figure S1c is a DNA sequence (meaning use T not U).

(3)	There are many gRNA sequences in which the first nucleotide is G and does not match the DNA sequence, such as Figure S1d. Though the authors described "the 20-bp complementary oligos, corresponding to each target site … were inserted" (Line 384 - 387), these gRNA and DNA sequences match only 19-bp. I suppose this G is the extra sequence for the transcriptional start point. In any case, the authors have to explain the detail of vector design and plasmid construction.

**Authors Response**

**Point-by-point responses to the reviewers’ comments:**

*Our response: First, we fully appreciate the reviewer’s constructive comments and suggestions that have allowed us to make our revised manuscript much better than the original.*

Reviewer #1: In the manuscript of Xu et al., the authors thoroughly tested the gene editing performance of SpCas9 variants, SpG and SpRY, and SpRY-derived base editors in transgenic rice and obtained the following findings: (1) SpG performs well on NGD PAMs but is less efficient than SpCas9-NG; (2) SpRY performs well on a broader range of PAMs than SpG, exhibiting a preference to NGD and NAN PAMs; (3) both SpRY-derived cytosine and adenine base editors can efficiently induce C-to-T or A-to-G conversions in the target sites with non-canonical PAMs; (4) SpRY can induce considerable self-editing events on sgRNA transgenes, whereas SpRY-derived base editors induce much less self-editing events. Overall, this is a timely and well-designed research that will have a broad impact on the genome editing practice in plant research and crop engineering. This reviewer only has some minor comments:

1. Line 143, a conjunction is needed right before 11.11%.

2. Line 151, Figure 2a, "di-allelic" should be "bi-allelic"?

3. Line 153, the sentence "being the ratios ... relatively higher" should be reworded.

*Our response: We appreciate the careful reading of the reviewer. Revisions were made based on the above suggestions.*

4. Lines 238-240, where to find the information about indel positions? Is there any reference to justify the reasoning that the indel can be induced by the deaminase?

*Our response: The sequence information containing the indel positions was showed in figure 3b (line #30). The cytidine deaminase deaminates cytidines (C) to uredines (U), and uracil DNA glycosylase (UDG) can excise the U in the C:U mismatch base pair to form an apyrimidinic (AP) site. Endogenous DNA-apurinic or apyrimidinic site lyase (AP lyase 2) cleaves AP sites and generates DNA nick at these sites. Then, these nicks are repaired by the NHEJ. As a result, these indels are located in the base-editing window (APOBEC3 induces mutations during repair of CRISPR-Cas9-generated DNA breaks, Nature Structural & Molecular Biology, 2018, 25, 45-52). In our earlier report, we described the frequent occurrence of indel mutations caused by hAID*Δ. By comparing rBE5 (hAID*Δ-Cas9n) and rBE9 (hAID*Δ-Cas9n-UGI) side-to-side, we found that the frequency of indels caused by hAID*Δ (located in the base editing activity window) was significantly lower than by rBE5, which suggested that these indels are resulted from the robust deaminase activity of hAID*Δ (Ren et al., Molecular Plant, 2018, 11(4): 623-626). Accordingly, based on the mechanism mentioned above, APOBEC-Cas9 fusion-induced deletion systems (AFIDs), which combine Cas9 with human APOBEC3A (A3A), UDG and apurinic or apyrimidinic site lyase, was developed (Wang et al., Nature Biotechnology, 2020, https://doi.org/10.1038/s41587-020-0566-4).*

5. Fig. 2, in some cases, the sgRNA-editing frequencies were extremely high, whereas the on-target editing frequencies were 0 or very low in the same transgenic rice. It is very likely that disruption of sgRNA sequences by self-editing has prevented on-target editing. The authors can highlight these data in Discussion between lines 302-305.

*Our response: We thank the reviewer for the valuable comments, it has been revised in the manuscript (Line 312-315, page 11).*

6. Fig. 3d and 3g, it is unusual that the base editing occurred in BSR-K1 skipped the two central C. Also, only C-to-G rather than C-to-T substitution occurred for the two outside C. It is worthy to mention this to readers.

*Our response: We thank the reviewer for their careful attention. We have added “an atypical” to line 254. As reported from our lab (Ren et al., Molecular Plant, 2018, 11(4): 623-626) and other lab (Ibrahim C. Kurt, Nature Biotech, 2020, https://doi.org/10.1038/s41587-020-0609-x), CBE, containing cytidine deaminases and UGI, generates C-to-T mutations in most time, but it causes low-frequency C-to-G or C-to-A mutations some time. We don`t know the reason behind it, maybe it’s locus-dependent.*

Reviewer #2: In this study, Xu et al. developed new plant genome editing tools using SpG and SpRY variants and showed that SpRY can expand the target scopes in genome editing of rice. They further described a novel cytosine and adenine base editors using the SpRY which can expand target selectivity for base editing. The authors also found that SpRY could target the sgRNA sequence in the T-DNA in transgenic plants. Overall, this study could provide useful tools for plant research and crop breeding. However, I have several concerns regarding the experimental design and results of the nuclease activity and PAM preference of SpRY.

1. I could not understand how authors evaluate mutation efficiency of transgenic calli.

For example, in Fig 1, 25/40 (62.5%) may indicate mutations were detected in 25 calli out of 40 calli. Then, 11 calli out of 25 calli were shown to be mono-allelic mutation.

However, I think each transformed callus should be chimeric and mono-allelic and bi-allelic mutations could be occurred in different cells in the same clump of callus.

*Our response: We thank the reviewer for this concern. In our lab, the fast rice transformation method has been using with immature rice embryos, small clump of resistant calli with independent transformation events are easy to be picked out, genotyped with mono- and bi-allelic mutations. In our experiences, the detected efficiency truly reflects the really one obtained by counting transgenic plants.*

2. For the targeted mutagenesis using SpRY, authors used two gRNAs per transformation construct. I'm afraid that this experimental design could not evaluate the editing activity and PAM preference of SpRY properly. Since, it has been reported that additional catalytically dead Cas9 binding at proximal locations improves Cas9 activity (Chen et al., Nature Communications, 2017; Liu et al., Genome Biology, 2019). As shown in Fig. 2, mutations were introduced into only one of the two targets in several genes. Mutation efficiencies could be affected by Cas9 bound to the other target site. Thus, it is inappropriate to conclude nuclease activity and PAM preference of SpRY on the basis of these mutation efficiencies. Authors have to show the mutation efficiency using a single sgRNA per transformation construct.

*Our response: Thank you for your critical assessment. As mentioned, it has been well documented that CRISPR-mediated genome editing and gene transcription regulation are somehow affected by chromatin states, chemicals, modification enzymes, even Cas/sgRNA which are related to chromatin remodeling have been utilized to enhance the genome editing efficiency. In our design, we have two gRNAs to target each endogenous gene to attenuate the effect of chromatin structure to some extent, therefore the recognition of different PAM sequences by SpRY in rice cells could reflect the actual PAM specificity. Alternatively, lots of plant researchers prefer using a single construct with 2 sgRNAs targeting a single gene of interest to generate the loss-of-function mutants in their studies. Therefore, we think that our experimental design is appropriate.*

3. The data in this manuscript showing the application of base editors using SpRY is very limited, especially cytosine base editors were evaluated with only NGC and NAG PAMs target sites. These experiments are insufficient to demonstrate the superiority of SpRY. The adenine base editors' experiments showed base substitutions in the NAA and NAT PAM target sites, demonstrating the advantages of using SpRY compared to the cytosine base editors' experiments. However, as described by the authors, the mutation efficiencies by SpRY greatly depend on the target locus. Therefore, more targets must be tested for each PAM and examined for base substitution rates.

*Our response: Thank you for your critical assessment. The superiority of SpRY is proven by the detailed assay on the broadened PAM compatibility of SpRY nuclease (Fig 2). Generally speaking, to address the property of novel base editors, we usually use the wild-type Cas proteins directly (i.e. xCas9, Cas9-NG, ScCas9, SpG, SpRY, Cas12), instead of base editors themselves, to answer the question on PAM specificity, because its nuclease activities are robust and easy to be tested. At the next step, we test the engineered base editor at representative PAM sites (novel PAMs) according to the results of Cas nucleases to confirm it works, so the base editing data are to address the compatibility of Cas proteins and nucleoside deaminases. In our experiences, base editing of a large number of target sites results in highly varying editing efficiencies, it is dependent on many factors (i.e. chromatin state, editing window, DNA context, deaminase-encoding genes used, etc.), but not on PAM recognition, these are another story.*

*A manuscript from Dr. Jinxiao Yang’s lab SpRY (in revising for Mol. Plant, personal communication) presents efficient base editing at other target sites in rice, it confirms the findings of our study. Anyway, the result of two additional target sites (OsGSK4 and BSR-D1) for adenine base editing was just obtained and we added it in fig 4.*

4. The authors found that SpRY not only targeted genome but also self-targeted sgRNA sequence in T-DNA in transgenic plants. However, according to the previous result of a high-throughput PAM determination assay (Walton et al., 2020, Science), SpRY seems to rarely recognize GTTT as a PAM. Therefore, it would be better to show in vitro cleave assay at GTTT PAM site, if possible.

*Our response: We respect the reviewer’s concern. Normally in vivo assay goes after in vitro assay. We have detected the self-editing events (indels induced by SpRY nuclease and nucleotide substitutions induced by hAID*Δ and Tad8e) in the T-DNA region in a total of 602 transgenic lines generated by 26 different sgRNAs. Similar results of Cas9-NG were also reported in rice by Dr. Pengcheng Wei (Qin et al., Nature Plant, 2020, 6(3):197-201) and by previous report (Ren et al., Mol Plant, 2019, 12(7):1015-1026). We believe that our data are solid and convincing. Anyway, we are about to perform the genome re-sequencing of some plants in T0, T1, and T2 generations to evaluate the off-target effects and PAM specificity of SpRY nuclease and its-derived base editors, the GCC PAM specificity of SpRY in planta will be investigated in more detail then.*

Other comments:

Line 59

"double-stranded break (DSB)" should be "DNA double-strand break (DSB)".

*Our response: Revisions were made based on the above suggestions.*

Line 63 - 68

I guess the description of the base editor in the background should be a bit more detailed.

*Our response: Thank you for your suggestion, we have added more information on CBE and ABE in this manuscript (Line 63-75, page 3).*

Line 279 - 280

Sretenovic and colleagues have already reported cytosine base editors and adenine base editors using iSpyMacCas9 which can recognize NAAR PAMs in rice (https://doi.org/10.1016/j.xplc.2020.100101). Hence, the authors' description "this is the first time a base editor which recognized non-G PAM sites in plants is described" is incorrect.

*Our response: Thank you for pointing this out, the sentence has been deleted.*

Line 335 - 336

As mentioned above, the number of targets examined in this manuscript is not sufficient to prove efficient base editing at minimal NA PAM site.

*Our response: The sentence has been deleted in this manuscript.*

Line 367

"adenine" should be deleted.

*Our response: As suggested, it has been deleted.*

Figure 3c

The sequence information appears to be wrong. The position of PAM sequence shown in the pale purple arrows is off, and target gene and base editor names and rBE66 are deleted.

*Our response: Thank you for pointing out this, it has been revised in the manuscript.*

Figure 3d

The sequence is opposite to the other figures (3g, 3h) and is not easy to see.

*Our response: As suggested, it has been revised in the figure.*

Figure 4f

Target region should be mentioned like Figure 3h.

*Our response: As suggested, the information has been added.*

Figure S1 - S18

These supplementary figures are fully incomplete.

(1) There are too many errors in target sequence information, such as Figure S1a (no-bold characters), S2c (misalignment of position).

*Our response: Thank you for pointing out. It has been revised.*

(2) In supplementary figures, the authors showed sgRNA sequences, but only Figure S1c is a DNA sequence (meaning use T not U).

*Our response: Thank you for pointing out. It has been revised.*

(3) There are many gRNA sequences in which the first nucleotide is G and does not match the DNA sequence, such as Figure S1d. Though the authors described "the 20-bp complementary oligos, corresponding to each target site … were inserted" (Line 384 - 387), these gRNA and DNA sequences match only 19-bp. I suppose this G is the extra sequence for the transcriptional start point. In any case, the authors have to explain the detail of vector design and plasmid construction.

*Our response: We thank the reviewer for their careful attention. All sgRNAs are 20 bp in size with a 5’ “G” as the transcriptional start site. These sgRNA and DNA sequences match 19 or 20 bp. It has been revised in the manuscript (Line 396-397, page 14).*

**Second round of review**

**Reviewer 1**

The authors have addressed my concerns during revision.

**Reviewer 2**

The authors have responded most, if not all, of my concerns in revised manuscript.

I have some very minor comments that was missed in the first review.

1. In figure legends of Fig.1, 3 and 4, "nuclear localization sequence" should be "nuclear localization signal".

2. In figure legend of Fig. 4, I guess TadA7.10 is not gene name. "the evolved E.coli TadA7.10 gene" should be "the evolved E.coli TadA gene" or "the evolved TadA7.10"?

**Authors Point-by-point Response to Reviewers**

Reviewer #2: The authors have responded most, if not all, of my concerns in revised manuscript and comment, and their responses are generally acceptable.

I have some very minor comments that was missed in the first review.

1. In figure legends of Fig.1, 3 and 4, "nuclear localization sequence" should be "nuclear localization signal".

*We thank the reviewer for their careful attention. It has been revised in the manuscript.*

2. In figure legend of Fig. 4, I guess TadA7.10 is not gene name. "the evolved E.coli TadA7.10 gene" should be "the evolved E.coli TadA gene" or "the evolved TadA7.10"?

*As suggested, “the evolved E.coli TadA gene” is mentioned in the manuscript.*
